# Targeted Covalent Photoswitch for Two-Photon Control of Endogenous Receptors

**DOI:** 10.1021/jacs.5c19954

**Published:** 2026-03-19

**Authors:** Ramona Santini, Galyna Maleeva, Rosalba Sortino, Santiago Pons-Allés, Cristian Ramos-Guerra, Carlo Matera, Pau Gorostiza

**Affiliations:** † Institute for Bioengineering of Catalonia (IBEC), The Barcelona Institute for Science and Technology, Barcelona 08028, Spain; ‡ CIBER-BBN, ISCIII, Barcelona 08028, Spain; § Doctorate Program in Organic Chemistry, University of Barcelona, Barcelona 08028, Spain; ∥ Catalan Institution of Research and Advanced Studies (ICREA), Barcelona 08010, Spain

## Abstract

The study of intact cells and their signaling circuits with light requires a stimulation strategy that is focused, deeply penetrating, and does not damage them. Implanted optic fibers, light-emitting diodes, and luminescent materials operated externally with tissue-penetrating infrared (IR) light are invasive or limited by light attenuation around the illumination point. To overcome these barriers, two-photon pharmacology takes advantage of femtosecond-pulsed IR laser light to produce deep and spatiotemporally precise cellular stimulation using specially designed photoswitchable drugs. Compounds that can be covalently tethered to the target neuroreceptor perform particularly well. However, the tethered photoswitches reported to date require mutagenesis of the target protein, which prevents the use of photopharmacology to stimulate the nervous system in wild-type animals. Here, we report the first two-photon optimized targeted covalent photoswitch (TCP_2P_) that combines the efficient two-photon isomerization of *ortho-*fluoro-substituted azobenzene with the ability to conjugate to nucleophilic residues of endogenous proteins (AMPA and kainate ionotropic glutamate receptors in neurons). TCP_2P_ is readily obtained by click coupling of two precursor compounds prior to use, and after simple incubation, it enables controlling neuronal activity at one- and two-photon excitation up to 800 nm without genetic modifications.

## Graphical abstract

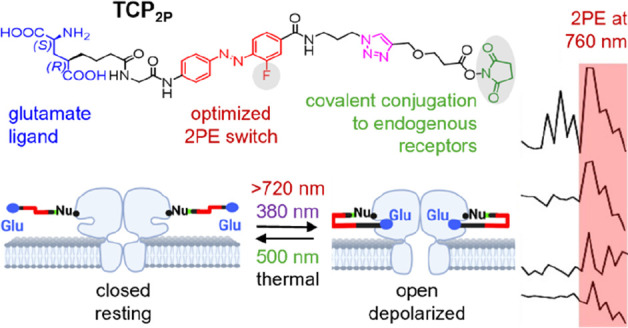

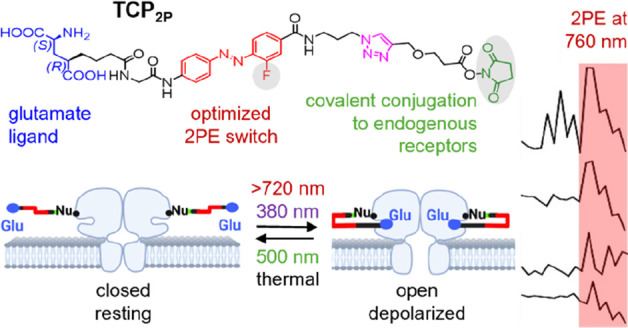

## Introduction

Photopharmacology relies on photoswitchable drugs to control the activity of proteins with light. It aims to deliver both therapeutic applications without adverse effects in nonilluminated regions and advanced research tools to investigate complex and dynamic cells and tissues, like neurons and neuronal circuits. Its ultimate goals, therapeutic or fundamental, cannot be fulfilled without illumination that is localized in target regions of interest such as specific tissues, organs, nerves, individual cells, or their compartments, like axons or spines in the case of neurons.

Methods of localized illumination include implanted optic fibers to convey light from external sources,^1^ implanted microfabricated light-emitting diodes (LEDs) with integrated batteries^2^ or radiofrequency links,^3^ and luminescent materials that are operated externally with tissue-penetrating infrared (IR) light.^4^ However, these methods are invasive; their “localization″ relies on the attenuation of light by the tissue surrounding the light source, or they simply neglect the fact that light is propagated around light emitters and all along light beams, activating in its way the photoswitchable drugs at unintended locations.

The basic development steps of photoswitchable drugs (to achieve high efficacy, potency, selectivity, drug-likeness, and light responsiveness) generally employ conventional light sources like continuous-wave LEDs, lasers, fluorescence, and halogen lamps.^5^ However, it can be argued that photopharmacology should include, by design, the means to allow truly three-dimensional (3D) localized activation of drugs if its therapeutic and fundamental goals are to be attained.

For that purpose, both tethered^6–8^ and free^9–11^ photoswitchable drugs have been operated with multiphoton excitation (MPE) using femtosecond-pulsed IR light,^12–14^ which penetrates through tissue without attenuation and, owing to the nonlinear dependence of MPE, allows to activate the drugs only at the focal spot (Figure 1a). In addition, covalently tethered photoswitches are not subject to diffusion of the unintended photoisomers. This convenient property, however, has only been demonstrated by tethering a cysteine-reactive photoswitch (e.g., based on maleimide^15^) to cysteine residues introduced in the receptor using mutagenesis or by tethering an overexpressed protein using a SNAP tag (“optogenetic pharmacology” or “optochemical genetics”).^16,17^ This methodological complexity contrasts with the MPE of endogenous (nonmutated) receptors enabled by freely diffusible photoswitches and prompts to demonstrate MPE in endogenous receptors using tethered compounds. Achieving MPE of photoswitches tethered to endogenous proteins using simple and biocompatible chemical means would offer an unprecedented “handle” to manipulate endogenous proteins with pharmacological, 3D, and temporal selectivity. Notably, a tool like this would allow us to observe the outcomes of the precise manipulations in their intact biological context.

**Figure 1:**
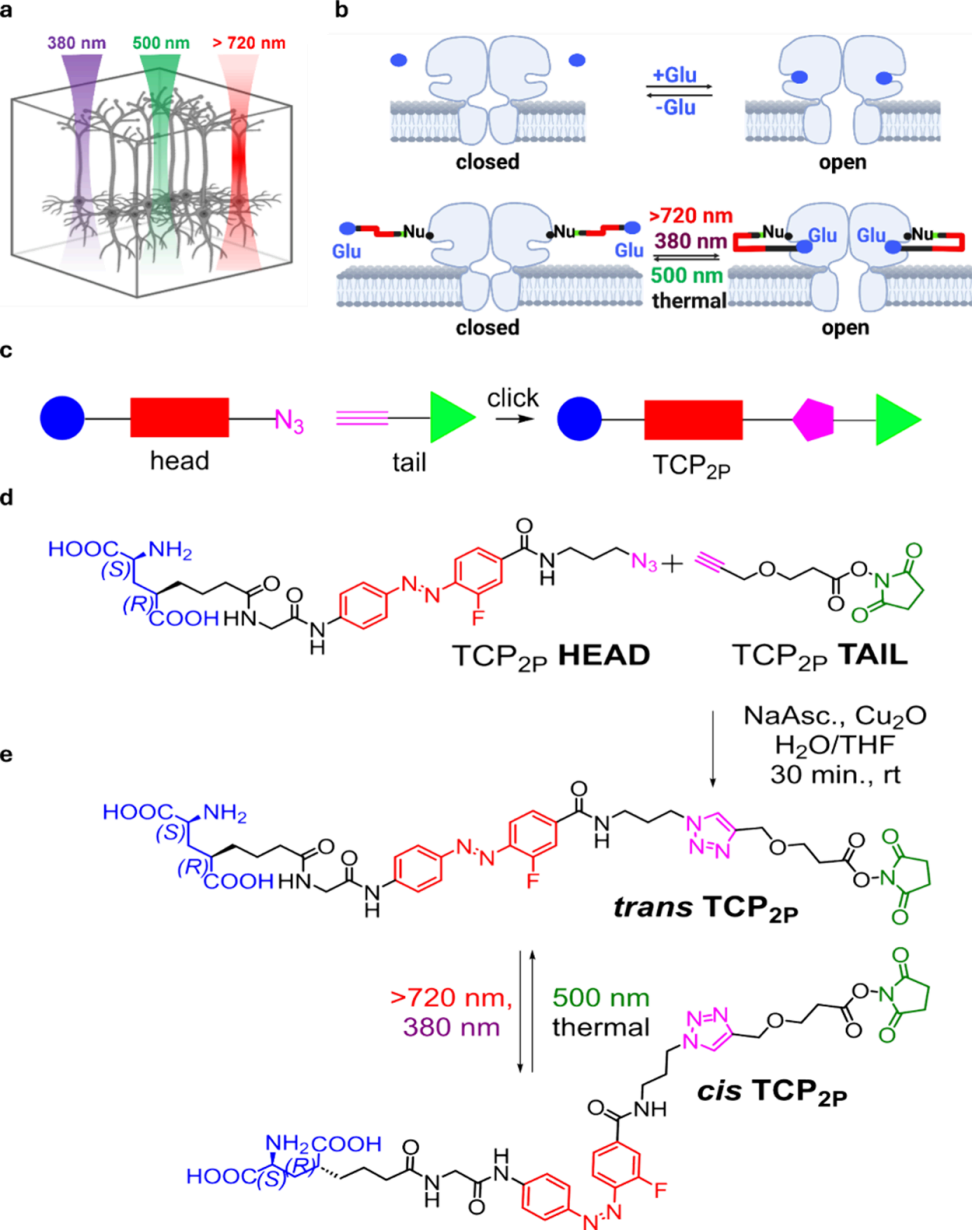
Purpose and design of two-photon optimized targeted covalent photoswitch TCP_2P_. (a) Diagram sketching the advantages of two- vs one-photon excitation (2PE, 1PE) to activate cortical neurons. 2PE (red, 720 nm) allows for deeper and focused stimuli owing to the tissue penetration of IR light and the enhanced absorption of pulsed laser light at the focal point. (b) Activation and channel opening of iGluRs mediated by glutamate (top) and by *trans* and *cis* TCP_2P_ (bottom; closed and open ion channel, respectively). 1PE (380 nm) and 2PE (>720 nm) induce *trans* to *cis* TCP_2P_ isomerization, allowing the glutamate moiety to the receptor site and subsequent opening of the cation-permeable channel and membrane depolarization. Channel activation is reverted back to *trans* under 500 nm or upon the thermal isomerization of TCP_2P_. (c) Schematic representation of the click coupling strategy used to obtain TCP_2P_. The glutamate ligand is highlighted in blue, the azobenzene photoswitch in red, the click reaction moieties in pink and the succinimide reactive anchoring group in green. (d) Molecular structures of the “head” and “tail” precursors of TCP_2P_ are freshly coupled via a copper­(I)-catalyzed azide–alkyne cycloaddition prior to incubation in neuronal tissue. (e) Chemical structure and photoisomerization of TCP_2P_.

To take advantage of tethered conjugation in endogenous glutamate receptors (GluRs), here we have combined the two-photon excitation (2PE)-optimized design of the MAG_2P_F_^slow^ photoswitch (*ortho* F-substituted azobenzene)^8^ with the protein conjugation approach of TCP9 to endogenous ionotropic glutamate receptors.^18^ Briefly, TCP9 bears a succinimidyl electrophilic reactive group that is coupled to a MAG-like photoswitch by Cu-catalyzed azide–alkyne cycloaddition^19^ and allows fast conjugation to nucleophilic residues in the endogenous glutamate receptor. The resulting 2PE-optimized analog of TCP9 (named TCP_2P,_ Figure 1b–e) can be incubated in cultured cells for few minutes and washed out, allowing it to reversibly activate untransfected hippocampal neurons with both continuous illumination (one-photon excitation, 1PE) and 2PE during hours. Thus, TCP_2P_ merges 2PE (tissue penetration and focalization), simplicity of use in endogenous receptors, and compatibility with biological preparations.

## Results and Discussion

The main drawback of using highly electrophilic anchoring groups (e.g., *N*-hydroxy-succinimide esters) for ligand conjugation to receptors is their tendency to react with nucleophilic groups contained in most ligands (intra- and intermolecular reactivity between the end groups of TCP_2P_ in Figure 1e). A convenient strategy to circumvent this problem is to split the self-reactive compound in two precursor molecules (Figure 1c): a ‘head’ bearing the active ligand and the photoswitchable moiety (glutamate and azobenzene in Figure 1d), and a ‘tail’ bearing the anchoring unit^18,20^ (NHS ester in Figure 1d). Using copper­(I)-catalyzed azide–alkyne cycloaddition (’click’),^21^ these precursors are readily coupled prior to cell incubation and conjugated immediately to the protein, to minimize unintended reactions.

The tail compound is commercial and bears the NHS group and the alkyne group necessary for coupling with the head. The head compound contains the reactive azide moiety, glutamic acid (a GluR ligand), and a 2PE-optimized photoswitchable azobenzene.^8^ This moiety contains weak electron-donating and electron-withdrawing mesomeric groups in *para* and a strong inductive group (−F) in *ortho* to the azo-bond. This allows a high 2PE cross section (σ), rendering it active with IR light without reducing the lifetime of the *cis* state.^8^

The optimal length of TCP9 after coupling head and tail is 28 bonds, calculated from the reactive carbonyl group to the C-4 of glutamate.^18^ In TCP_2P_, it was necessary to insert a glycine unit in *para* to the azobenzene core to enhance reactivity (see below), resulting in an overall 31 bond length. As shown in Scheme 1, the final head is afforded by coupling **Module 1** (compound **11**, containing the azobenzene core and the azide reactive unit) and **Module 2** (compound **15**), which is a cyclic and protected derivative of glutamate originally developed by Volgraf et al.^15^

**Scheme 1:**
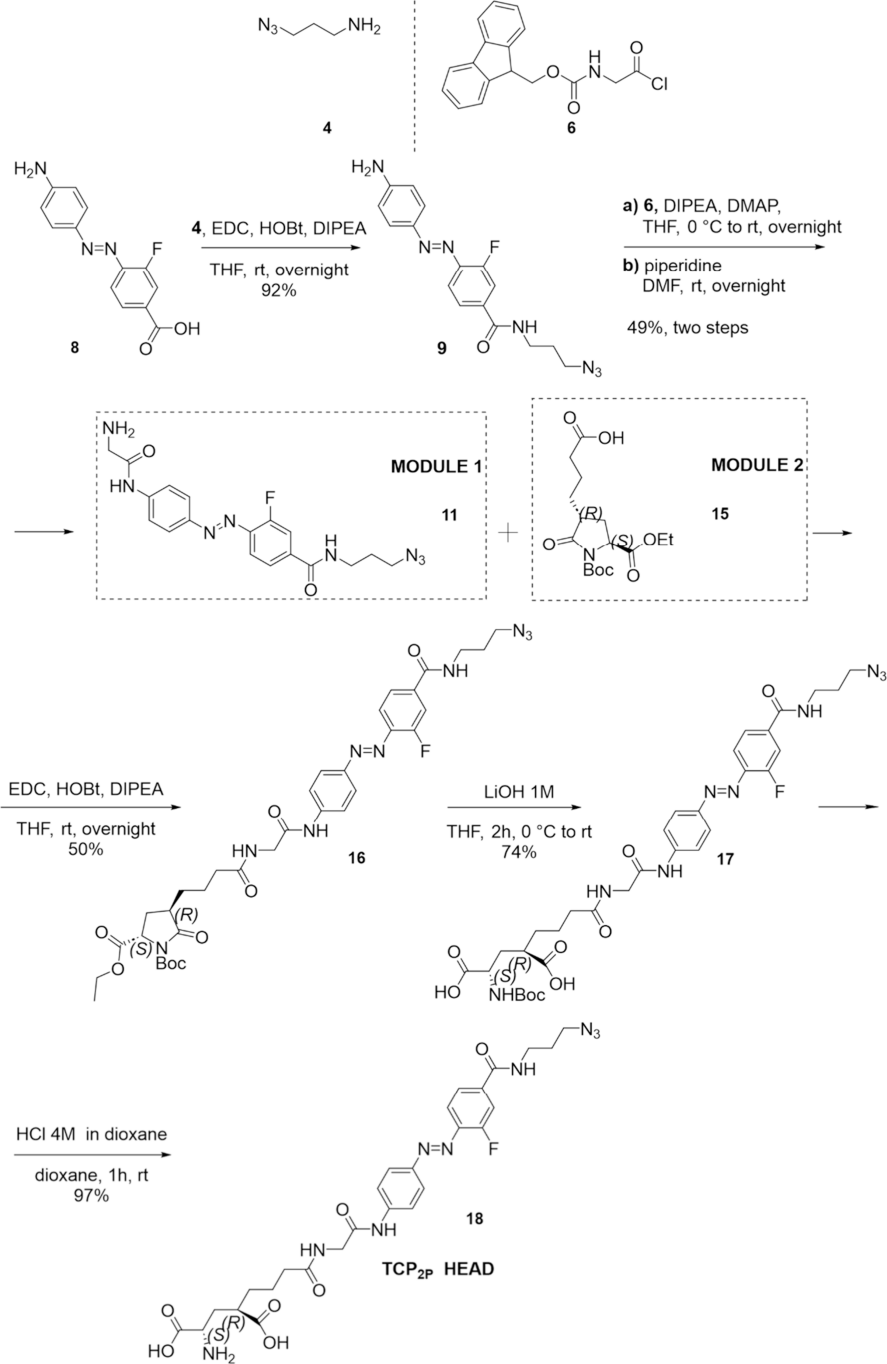
Chemical Synthesis of the TCP_2P_ Head (**18**), Bearing the Glutamate Moiety, the Photoswitchable Unit, and the Terminal Azide for Click Coupling^a^

a See full details in Scheme S1 (SI, same numbering of compounds).

The preparation of **11** started with the synthesis of the azobenzene core as described by Cabré et al.^8^ The diazotization of aminobenzoic acid **7** was followed by coupling with sodium phenylaminomethanesulfonate, which was readily available from aniline and the formaldehyde sodium bisulfite adduct. The following removal of the amino protecting group delivered amino acid **8** in 34% yield (see Scheme S1 in the Supporting Information (SI) for a detailed synthesis). This was coupled to amide **4**, which carries the azide group for the click reaction. The following steps consisted of the insertion of a glycine unit on the low-reactive aniline **9** by Fmoc-Gly-OH and −Fmoc group removal under basic conditions. Thanks to the aliphatic primary amine on glycine, intermediate **11** is more reactive than its precursor **9,** and this facilitates the coupling with intermediate **15** (**Module 2**). Once **Modules 11** and **15** were prepared, they were coupled through amide bond formation, followed by ring opening and removal of the -Boc group under acidic conditions. The final deprotection was carried out at a small scale, producing 8.7 mg of the final head compound **18** in quantitative yield and without the need of chromatographic purification. Compound identity and purity were assessed by NMR, HPLC-MS, and HRMS (SI).

To photochemically characterize the TCP_2P_ head compound **18**, absorption spectra were recorded with a UV–visible spectrophotometer using a 50 μM solution in PBS with 2% DMSO. As shown in Figure S24a,b, the best photoconversion from *trans* to *cis* was achieved by irradiating **18** with 380 nm light for 5 min. The more thermodynamically stable *trans* isomer was recovered by irradiating **18** for 5 min with 500 nm light or thermal relaxation. The thermal back relaxation kinetics were studied by irradiating the cuvette with 380 nm light for 5 min and recording the absorbance at 340 nm in the dark every 15 min, which yielded a lifetime of 98 min (Figure S24c). These results are similar to TCP9, whose photoconversion was also obtained by irradiating with 380 nm light for 5 min, and whose *cis* isomer showed a lifetime of 84 min.^18^ The TCP_2P_ head back isomerizes from *cis* to *trans* in the dark with a lifetime of 98 min, confirming the high thermal stability of the *cis* conformation that was previously observed computationally and experimentally.^8^ The lifetime of the TCP_2P_ *cis* isomer head confirms that inserting weak electron-donating and electron-withdrawing groups in *para* and a fluorine group in *ortho* to the azobenzene core yields a push–pull, slow-relaxing switch for 2PE.

The rate of photoconversion was studied by HPLC-PDA-MS, measuring the change in the integrated area of the peaks corresponding to the *trans* and *cis* isomers at the isosbestic point (298 nm) at dark equilibrium (90% *trans* and 10% *cis*) after irradiating with 380 nm light (38% *trans* and 62% *cis*) and after irradiating with 500 nm light (72% *trans* and 28% *cis*). Data are shown in Figure S25. The TCP_2P_ head shows lower percentages of photoconversion compared to MAG_2P_F_^slow 8^ (70% *cis* ^PSS^ under 380 nm and 85% *trans* ^PSS^ under 500 nm), but it is otherwise similar and includes an F in *ortho* for two-photon activation.

TCP_2P_ was obtained by coupling the head (**18**) and the commercially available tail (propargyl-*N*-hydroxysuccinimidyl ester, **19**, CAS: 1174157-65-3, Figure 1d and Scheme S2) using a ‘click’ version of the Huisgen azide–alkyne 1,3-dipolar cycloaddition reaction.^19,22^ In particular, to a solution of the head (1 eq, 0.7 mg, 1.14 μmol), Cu_2_O (2.40 eq, 0.4 mg, 2.74 μmol), and sodium ascorbate (4 eq, 0.9 mg, 4.56 μmol) in 30 μL of Milli-Q water was added a solution of the tail (1.10 eq, 0.3 mg, 1.25 μmol) in THF (11 μL), and the reaction was vortexed for 30 min at room temperature. The mixture was diluted to 10 mM DMSO and used immediately or stored at −80 °C for later use.

We implemented a quality control check of each click coupling reaction by HPLC-PDA-MS, as shown in Figures S26a,b and S28 (SI). Within 10 min of coupling TCP_2P_ head and tail and before proceeding with biological experiments, we analyzed the supernatant of the reaction crude to confirm the identity of the main peak as intact and reactive TCP_2P_ product and to characterize the reaction byproducts. These generally included TCP_2P_ derivatives with hydrolyzed (unreactive) tail and/or modified glutamate due to electrophilic attack, which are not suitable for receptor conjugation and can compete with the affinity labeling process^18^ (Figure S27).

To test the ability of TCP_2P_ to photoswitch neuronal activity, we freshly coupled the head and tail precursors as described above and incubated glass coverslips with cultured hippocampal neurons at a 200 μM concentration (1.3% DMSO, 0.2% THF) for 3–4 min at pH 9, followed by washout in an extracellular solution of pH 7.4. First, we tested the responses to one-photon excitation (1PE) using whole-cell patch clamp electrophysiological recordings under 380 or 500 nm light using a monochromated xenon lamp (Polychrome V, Till Photonics). The results are shown in Figure 2a,b and control recordings in Figure S29. TCP_2P_ elicited robust neuronal depolarization and firing under 380 nm light (frequency increased to 2.2 ± 0.4 Hz, *n* = 11) that was fully reversible under 500 nm illumination, indicating a high efficacy of receptor conjugation and photocontrol of activity (Figure S30).

**Figure 2:**
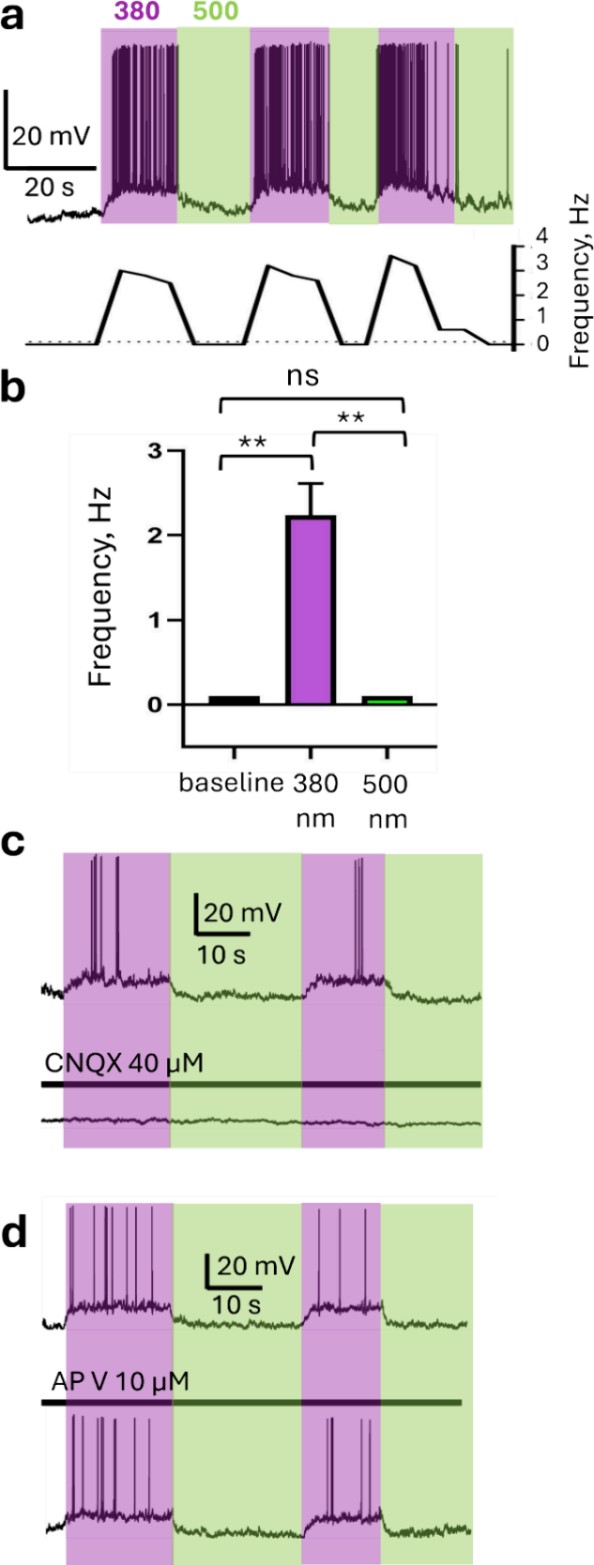
Incubation of cultured hippocampal neurons in TCP_2P_ allows photocontrolling their firing activity. (a) Whole-cell electrophysiological recording of a cultured hippocampal neuron in voltage clamp mode, *V*_hold_ = −60 mV. After TCP_2P_ incubation for 4 min and washout, neuronal firing is elicited by 380 nm light (indicated by the violet box) and reversed fully and rapidly by green light (500 nm, indicated by the green box). No significant differences were observed between darkness and 500 nm light. The graph below the trace shows the neuronal firing frequency as a function of time. (b) Quantification of the firing frequency of neurons incubated in TCP_2P_ before illumination (black bars) and during illumination with 380 and 500 nm light (violet and green bars, respectively). Data are represented as mean ± SEM, *n* = 11 cells. The statistical significance of the differences between the groups was evaluated using the Friedman test, ** *P* ≤ 0.01, ns – not significant. (c) Representative recordings of a neuron incubated in TCP_2P_ in the absence (top panel) and in the presence (bottom panel) of the GluA and GluK antagonist CNQX (40 μM). CNQX blocks the ability of TCP_2P_ to photoinduce neuronal firing or even membrane depolarization. (d) Photoresponses of a TCP_2P‑_incubated neuron in the absence (top) and in the presence (bottom) of the GluN antagonist APV (10 μM), which does not inhibit the photocontrol of neuronal firing.

To identify the glutamate receptor type targeted by TCP_2P_, we used competitive antagonists that have been shown to inhibit GluA, GluK, and GluN activity in cultured neurons and to abolish excitatory neurotransmission.^23,24^ Regardless of their amplitude (conjugation efficacy), photoresponses were blocked by 40 μM CNQX (GluA and GluK receptor antagonist, Figure 2c) but not by 10 μM of APV (GluN receptor antagonist, Figure 2d), suggesting that TCP_2P_ conjugates preferentially to endogenous GluA and GluK receptors, as in the case of TCP9.^25^ Such a preference suggests that the ability of TCP_2P_-incubated neurons to retain their membrane potential and to fire action potentials may be related to low unspecific conjugation of the NHS ester group to nucleophilic residues in other membrane proteins, which would explain its apparent lack of acute cytotoxicity. Interestingly, TCP_2P_ photoresponses are inhibited by 25-fold lower antagonist concentrations than TCP9, which requires 1 mM DNQX in overexpressed GluK_1._ ^18^ The empirically obtained values of 40 μM and 1 mM antagonist concentration inform about the local effective concentration of tethered glutamate “seen” by the binding site for TCP_2P_ and TCP9, respectively. This difference may be partially due to the 5-fold higher potency of CNQX used with TCP_2P_ compared to DNQX for TCP9, but also to the 3-bond longer tether of TCP_2P_, which increases the volume sampled locally by its glutamate moiety around the binding site in the receptor, thus reducing its local effective concentration compared to TCP9 and MAG.^26^

We further characterized TCP_2P_ photoresponses using calcium imaging to obtain simultaneous recordings from several neurons and to allow using a fluorescence microscope equipped with 2PE. Prior to TCP_2P_ conjugation, cells were incubated in a cell-permeable calcium-sensitive fluorescent dye (OGB-1AM, Life Technologies) and washed out with extracellular medium. Fluorescence images were acquired as a function of time in an inverted fluorescence microscope coupled to the monochromator for illumination (excitation 488 nm; emission 510 nm). In this setup, TCP_2P_ isomerization was controlled by whole-field illumination with 380 and 500 nm light (1PE). Example traces corresponding to the calcium-dependent fluorescence intensity of individual neuronal somas and their quantification are displayed in Figure 3a. They show robust calcium entry under violet light in about 1 min and partial depletion under green light, which is probably limited by the large amount of cytoplasmic calcium that must be evacuated after TCP_2P_ deactivation. On average, the fluorescence ratio *F*/*F*_0_ increased from 1 ± 0.003 to 1.20 ± 0.06 under 380 nm light and decreased to 1.11 ± 0.02 under 500 nm light (*n* = 11, Figure 3b). These results agree with the depolarizing photoresponses elicited by TCP_2P_ on GluRs (Figure 2a,b, Supporting Information Video 1), which may activate voltage-dependent calcium channels, thereby amplifying the calcium uptake by the cells.

**Figure 3:**
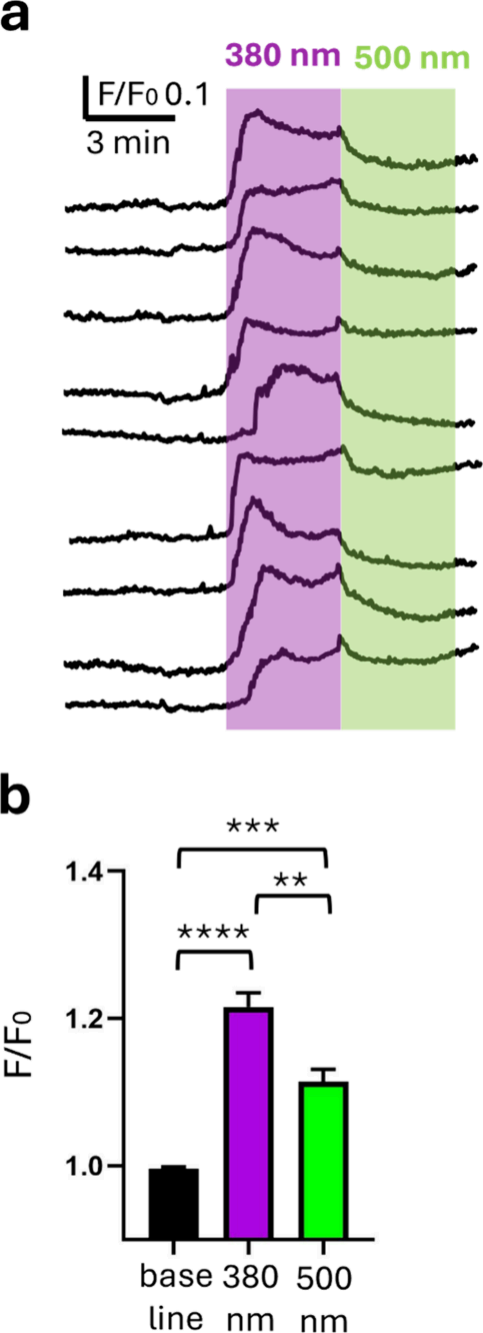
Incubation of cultured hippocampal neurons in TCP_2P_ allows photocontrolling their intracellular calcium concentration. (a) Representative traces from individual neurons in a fluorescence imaging recording of intracellular calcium concentration (OGB-1AM) in TCP_2P_-incubated hippocampal cells. Imaging pulses (100 ms every 2 s; see Supporting Information) were chosen to not interfere with photoisomerization. Longer pulses of 380 nm light (1.5 s every 2 s for 3 min, violet box) produce an increase in intracellular calcium concentration that is inhibited by 500 nm light (1.5 s every 2 s for 3 min, green box). The partial recovery of the photoresponses is likely due to the long 380 nm stimulations used in this case (3 min) that produce a calcium influx into the neurons that is greater than the ability of their ion pumps to restore normal intracellular calcium levels (compare to the full and rapid recovery of the neuronal membrane potential after 10–20 s photostimulation used in patch clamp experiments (Figure 2). (b) Quantification of fluorescence intensity before illumination (black bar) and during 380 and 500 nm illumination (violet and green bars, respectively). Data are represented as mean ± SEM, *n* = 9 cells. Statistical significance between groups was determined using a one-way Anova test, ** *P* ≤ 0.01, *** *P* ≤ 0.001, and **** *P* ≤ 0.0001.

To evaluate the photoresponses to 2PE, we took advantage of the calcium photoresponses obtained previously (Figure 3) and used a confocal microscope equipped with a tunable femtosecond pulsed laser, which allows one to photoisomerize TCP_2P_ with 2PE at different wavelengths (720–840 nm).^8,9^ Imaging was performed at 488 nm 1PE excitation and 510 nm emission. After TCP_2P_ incubation and washout, hippocampal neurons displayed a rapid and reversible increase in calcium fluorescence upon 2PE, as shown in Figure 4a for 760 nm (Supplementary Video 2). The fluorescence intensity ratio (*F*/*F*_0_) rose in about 10 s from 1 ± 0.006 to 1.40 ± 0.03 (*n* = 37) under 760 nm light, which is faster and higher than the 1PE responses (Figure 3). The 2PE calcium increase was more transient than with 1PE but repeatable and reproducible in different neurons (note that transient 2PE responses with MAG_2P_F_^slow^ have been reported in neurons compared to steadier responses in cell lines overexpressing GluRs,^8^ but the reason for this difference is unknown). Similar and even higher photoresponses were observed at 720 and 800 nm, respectively, whereas 840 nm did not elicit a significant calcium increase. Thus, the 2PE action spectrum of TCP_2P_ (Figure 4b) is like that of MAG_2P_F_^slow^ compound^8^ but allows using shorter wavelengths (720 nm, Figure S31), which may be convenient for certain applications and laser specifications.

**Figure 4:**
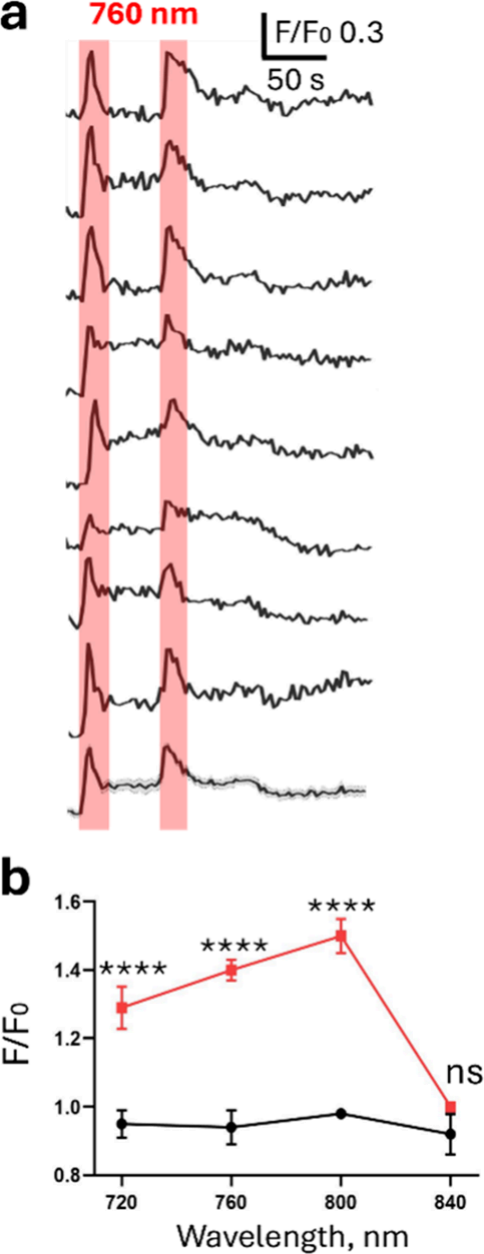
2PE transiently increases the intracellular calcium concentration of primary hippocampal neurons incubated with TCP_2P._ (a) Representative traces of calcium-dependent fluorescence (OGB-1AM sensor) recorded from individual neurons incubated with TCP_2P._ Red rectangles indicate the duration of 760 nm 2P illumination. (b) Dependence of the intracellular calcium increase (relative fluorescence) as a function of the 2PE wavelength in control (black line) and after TCP_2P_ incubation (red line)_._ Data are represented as mean ± SEM, *n* = 8–37 cells for each wavelength. Statistical significance between the control and TCP_2P_ incubated samples was determined using the Mann–Whitney test; **** *P* ≤ 0.0001, ns – not significant.

In most calcium imaging experiments, photoresponses were higher in the somas and synchronous with light stimuli. Interestingly, in some experiments displaying relatively low somatic photoresponses and overall fluorescence intensity, we noticed that activity was elicited mostly at isolated spots of micrometric size, as shown in Figure 5. These photoresponses can be attributed to synapses or dendritic branches producing subthreshold activation of the neuron. Spinal and dendritic photoactivation agree with the known expression and function of calcium-permeable iGluRs in postsynaptic compartments.^27–29^ These results prompt the application of TCP_2P_ and 2PE to investigate the dynamic properties of individual synapses and the integration of dendritic input signals in neurons.

**Figure 5:**
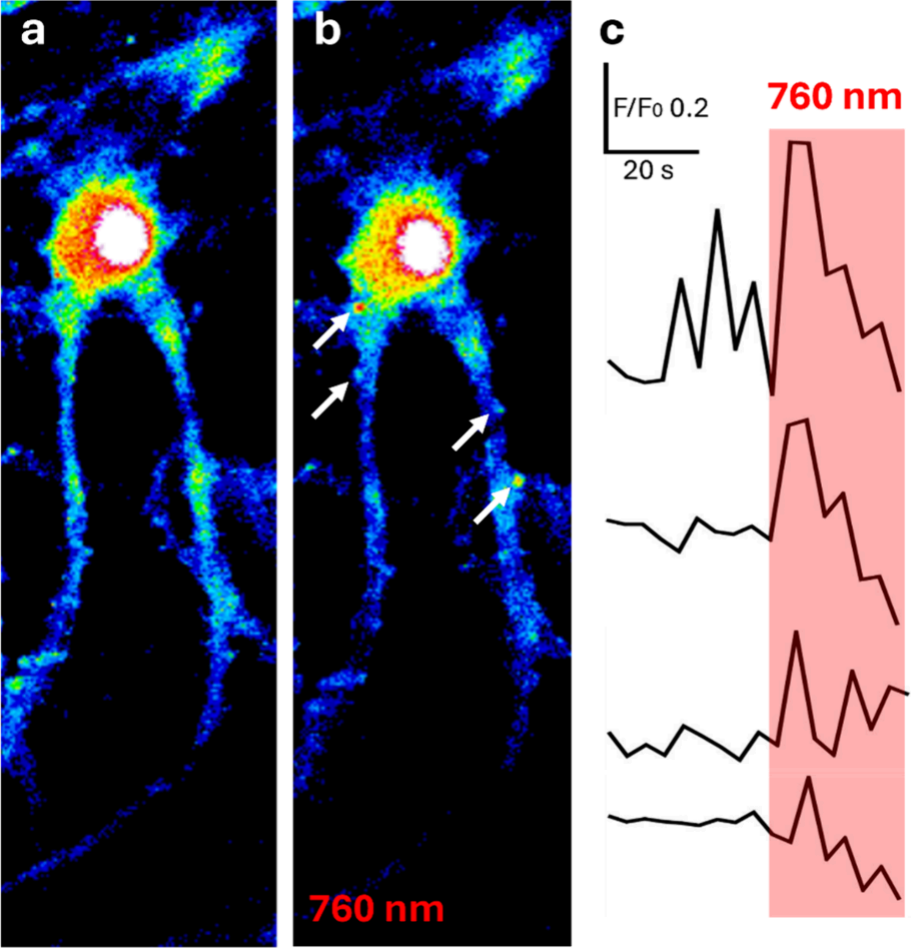
2PE of TCP_2P_-incubated neurons induces calcium responses in separate neuronal compartments that can be attributed to spines. Fluorescence images (OGB-1AM calcium sensor) of TCP_2P_-incubated hippocampal neurons before and during 2PE at 760 nm (a and b, respectively). (c) Traces of calcium-dependent fluorescence intensity in the regions of interest are indicated by white arrows in panel b. 2PE is indicated by the red rectangle.

Importantly, compared to other photostimulation methods like uncaging and optogenetics, TCP_2P_ has the unique advantages of covalent conjugation to endogenous receptors (long-lasting ability to photoswitch them after one-time conjugation and wash; tracking labeled receptors) combined with those of 2PE (deep penetration and μm-scale focalized stimulation in three dimensions). In contrast to 1PE-responding MAG and TCP9, which activate glutamate receptors in all neurons being crossed by the laser beam path, 2PE of TCP_2P_ enables activation of only neuronal compartments in the focal plane and avoiding those in planes above and below. Thus, TCP_2P_ is more suitable for studying the synaptic strength of isolated spines and dendritic branches and to obtain 3D-maps of endogenous receptor activity, especially in combination with red or IR activity reporters. In addition, the ability of TCP_2P_ to photocontrol the activity of endogenous receptors offers methodological simplicity and physiological relevance greater than those of MAG_2P_F_^slow^. Finally, the apparent lack of toxicity of TCP_2P_ in neurons encourages future applications in brain slices and *in vivo*.

The 2PE-optimized *ortho*-fluoro azobenzene switch reported in MAG_2P_F_^slow^ has been successfully introduced in another photoswitchable drug (TCP9), demonstrating the versatility of this motif to recondition existing photopharmacology for 2PE. Although the azobenzene group of ionotropic glutamate receptor photoswitches does not participate in ligand binding and thus tolerates bulky substituents,^30^ the minimal size and hydrophobicity of the fluorine substitution are likely to be also compatible with azolog designs^31^ where the azobenzene group is part of the pharmacophore, and even to improve their potency. Other 2PE approaches have been recently developed beyond azobenzenes.^14^

MPE raises radically new opportunities for photopharmacology.^12,13,32^ Although 3PE offers deeper penetration and higher resolution than 2PE,^33,34^ 3PE microscopes are still large, scarce, and costly. In contrast, commercial 2PE setups are available in shared research facilities and in certain specialized laboratories. Furthermore, recent breakthroughs in the miniaturization of pulsed lasers^35^ allow to envisage integrated, low-power, and inexpensive components equivalent to LEDs. In view of these advances and the enormous possibilities that they open for basic and therapeutic applications, it is necessary to develop and characterize new 2PE-optimized photoswitchable drugs for the truly localized and on-demand stimulation of endogenous cellular activity.

## Conclusions

In conclusion, we rationally designed and synthesized TCP_2P_, merging the best features of TCP9 and MAG_2P_F_^slow^. TCP_2P_ encompasses in a single compound two outstanding properties for neurobiology: (1) its abilities to conjugate to endogenous receptors without genetic manipulation and to control their activity with light and (2) the use of pulsed near-infrared light, which allows deep tissue penetration as well as focalized stimulation in three dimensions at the micrometer scale. TCP_2P_ can robustly modulate neuronal activity, as shown by patch clamp and calcium imaging at 1PE and 2PE. Multiphoton pharmacology overcomes the limits of low tissue penetration and tissue damage of traditional 1PE photopharmacology, and TCP_2P_ takes a leap forward to develop two-photon activated molecules to track and control the activity of endogenous receptors in intact tissue *in vivo* and studying complex neural networks and their role in diseases.

## Supplementary Material







## Data Availability

The data sets generated and analyzed during the current study are available from the corresponding authors on reasonable request.

## Notes

Synthesis, structural and photochemical characterization of TCP_2P_ HEAD and final TCP_2P_; additional experimental methods, including primary culture of hippocampal neurons, electrophysiological recordings, calcium imaging assays, and TCP_2P_ administration and control data (PDF)
Recordings of the response of hippocampal neurons incubated with TCP_2P_ to 1PE and monitored by Ca^2+^ imaging (AVI)
Recordings of the response of hippocampal neurons incubated with TCP_2P_ to 2PE and monitored by Ca^2+^ imaging (AVI)

## %

Department of Pharmaceutical Sciences, University of Milan, Milan 20133, Italy

## #

Enantia, Barcelona 08028, Spain.
