# Supplementary material for: Targeted Covalent Photoswitch for Two-Photon Control of Endogenous Receptors
Source: J Am Chem Soc. 2026 Mar 19;148(12):12708–16. doi: 10.1021/jacs.5c19954 (PMC13047687; doi:10.1021/jacs.5c19954)
Supplement: Supplementary file 1 [file ja5c19954_si_001.pdf]

SUPPORTING INFORMATION

Targeted covalent photoswitch for two-photon control of endogenous receptors

Ramona Santini,[1,2,3,+] Galyna Maleeva,[1,2,+] Rosalba Sortino,[1,2] Santiago Pons-Allés,[1,#] Cristian Ramos-Guerra[1], Carlo Matera,[1,2,%] Pau Gorostiza[1,2,4,*]

1. Institute for Bioengineering of Catalonia (IBEC), The Barcelona Institute for Science and Technology, Barcelona 08028, Spain.

2. CIBER-BBN, ISCIII, Barcelona 08028, Spain.

3. Doctorate Program in Organic Chemistry, University of Barcelona, Barcelona 08028, Spain.

4. Catalan Institution of Research and Advanced Studies (ICREA), Barcelona 08010, Spain.

+ Equivalent contribution

% Present address: Department of Pharmaceutical Sciences, University of Milan, Milan 20133, Italy.

# Present address: Enantia, Barcelona 08028, Spain.

* Correspondence. E-mail: pau@icrea.cat

Contents

SUPPORTING INFORMATION 1

General methods and materials for chemical synthesis and physicochemical characterization 3

Synthetic protocol for the preparation of TCP_{2P} HEAD 4

Photochemical characterization 31

Synthetic protocol for the preparation of TCP_{2P} 33

Outcome of click reactions 33

Primary culture of hippocampal neurons 36

Electrophysiological recordings 37

Calcium imaging 37

TCP_{2P} administration and photostimulation in live cell imaging experiments 37

Drugs 38

Data analysis 38

Abbreviations 41

Bibliography 44

General methods and materials for chemical synthesis and physicochemical characterization

All reagents and solvents were purchased from Sigma-Aldrich or Activate Scientific and were used without any further purification. TLC analyses were performed on commercial silica gel 60 F254 aluminum foils (Merck) and visualized under 254 and/or 365 nm lamp. Flash column chromatography: purifications were performed on a Biotage SP1 Flash Chromatography system, PanReac AppliChem silica gel 60 (40-63 microns) was used as stationary phase in direct phase and Biotage Sfar C18D in reverse phase; mobile phases are specified for each molecule. Nuclear magnetic resonance: spectra were registered with a Varian Mercury 400 MHz and a Bruker 400 MHz instruments. Chemical shifts (δ) are reported in parts per million (ppm) against the reference compound tetramethylsilane using the signal of the residual non-deuterated solvent [Chloroform-d δ = 7.26 ppm (1H), δ = 77.16 ppm (13C); Dimethylsulfoxide-d_6 δ = 2.50 ppm (1H), δ = 39.52 ppm (13C); Methanol-d_4 δ = 3.31 ppm (1H), δ = 49.00 ppm (13C); Water-d_2 δ = 4.79 ppm (1H), δ = 49 ppm (13C)]. Spectra were analyzed using MestreNova v14.2.0-26256. High-performance Liquid Chromatography: for synthesis and characterization of intermediates **2-18** measurements were recorded on a Waters Alliance e2695 separation module coupled with a Waters 2998 Photodiode Array Detector (190 – 800 nm) and a QDA Detector Acquity, with MassLynx software for data acquisition. Column: XSelect CSH C18 (3.5 µm, 4.6x50 mm). Column temperature was kept at 50 °C. Mobile phase: water w/0.1% HCOOH (solvent A) and acetonitrile w/0.1 % HCOOH (solvent B). Elution method: flow of 1.6 mL/min, runtime of 5 min; 0.0-3.5 min, 5-100 % B; 3.5−4.5 min, 100 % B. Data are reported as mass-to-charge ratio (m/z) of the corresponding positively charged molecular ions. Reported retention times are relative to the *trans* isomers. UV spectrophotometer: Photochromic behaviour of **TCP$_{2P}$ head** solution was examined recording UV/vis absorption spectra with a Shimadzu UV-1800 UV-VIS Spectrophotometer with standard quartz cuvettes (10 mm light path). The UV light sources were custom-made LEDs with wavelength of 365, 380, 400, 420, 430, 460 and 500 nm. Irradiation was conducted within a custom-made light/tight enclosure, ensuring controlled experiment conditions. The sample was positioned at a fixed distance of 5 cm from the light source. Irradiance of the LEDs: 380 nm, 0.51 mW/cm^2; 400 nm, 0.84 mW/cm^2; 420 nm, 1.40 mW/cm^2; 430 nm, 1.72 mW/cm^2; 460 nm, 1.48 mW/cm^2, 500 nm, 0.83 mW/cm^2. Measurement of the irradiance was conducted with a Thorlabs PM101 PD/TH optical power meter equipped with a Thorlabs S120VC sensor, with a diameter of 9.5 mm. All graphs and fitting were produced with GraphPad Prism version 9.2.0. High Resolution Mass Spectrometry: analyses were performed with a Thermo Scientific™ Orbitrap Fusion™ Lumos™ Tribrid™ with NanoESI positive ionization. Sample (2.46 mM **TCP$_{2P}$HEAD** solution in DMSO) was diluted 1/100 with ACN/H_2O/formic acid (50:50:1) for MS analysis. The sample was introduced by direct infusion (Automated Nanoelectrospray). The NanoMate (Advion BioSciences, Ithaca, NY, USA) aspirated the samples from a 384-well plate (protein Lobind) with disposable, conductive pipette tips, and infused the samples through the nanoESI Chip (which consists of 400 nozzles in a 20x20 array) towards the mass spectrometer. Spray voltage was 1.70 kV, delivery pressure 0.50 psi and m/z range 200-2000 a.m.u. Data was acquired with Xcalibur software, vs 4.2.28.14 (ThermoScientific). Elemental composition from experimental exact mass monoisotopic value was obtained with a dedicated algorithm integrated in Xcalibur software. Data are reported as mass-to-charge ratio (m/z) of the corresponding positively charged molecular ion.

Synthetic protocol for the preparation of TCP_{2P} HEAD

Compounds **2**, **4** and **8** were synthesized according to literature[1–3] Compound **15** was synthesized following the procedure previously described[4] . Azobenzene **8** was coupled through amide coupling reaction with **4**, which carries the azide group for the *click* reaction. The following steps consisted in the insertion of a glycine unit on the low reactive amino group of the azobenzene core followed by Fmoc- deprotection in basic conditions. The aliphatic primary amine on glycine makes intermediate **11** more reactive than precursor **9** and this allowed the coupling with pyroglutamate **15**, which was then hydrolyzed and saponified affording **17**. The final *tert*-butoxycarbonyl protecting group removal under acidic conditions converts intermediate **17** into **TCP_{2P} head (18)**.

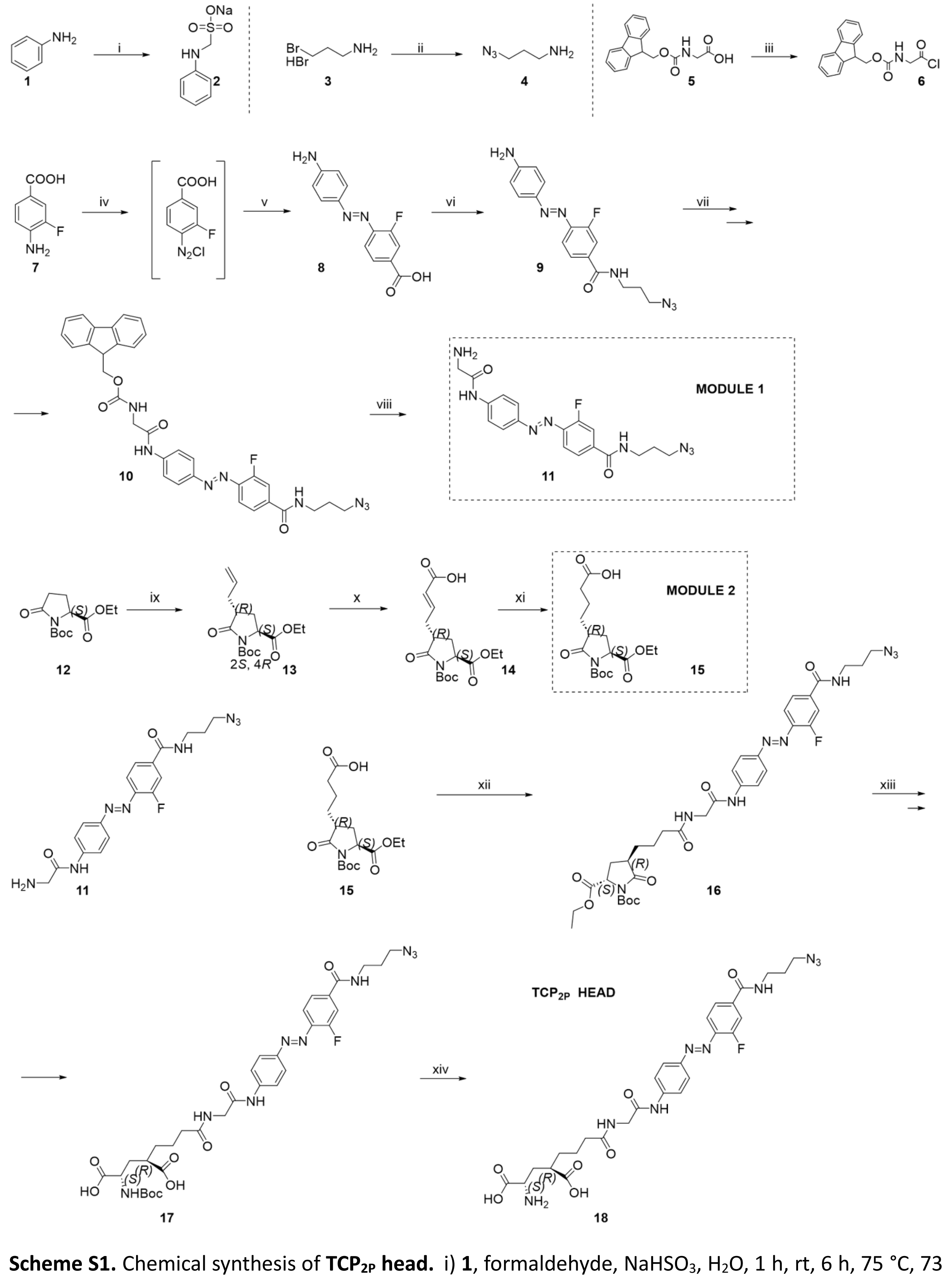

Scheme S1. Chemical synthesis of **TCP_{2P} head.** i) **1**, formaldehyde, $NaHSO_3$, H_2O, 1 h, rt, 6 h, 75 °C, 73 %; ii) a) **2**, NaN_3, H_2O, reflux, overnight; b) KOH, 71 %; iii) **5**, oxalyl chloride, cat. DMF, THF, 1 h, 0 °C, inert atmosphere; iv) **7**, HCl 5.5 M, $NaNO_2$, 0 °C; v) a) **2**, NaOAc 0.86 M, 0 °C, overnight b) NaOH 1 M, 4 h, 90 °C; c) HCl 5.5 M, 34 %; vi) **8**, **4**, EDC, HOBt, DIPEA, THF, rt, overnight, 92 %; vii) **9**,**6**, DIPEA, DMAP, THF, 0 °C to rt, overnight; viii) **10**, piperidine, DMF, rt, overnight, 49 %, two steps; ix) **12**, allyl bromide, LiHMDS 1 M, THF, 3 h, -78 °C, inert atmosphere, 23 %; x) **13**, acrylic acid, Grubb II generation catalyst,

DCM, reflux, overnight, 55 %; xi) **14**, H_2, Pd, MeOH, 3 h, rt, 77 %; xii) **11**, **15**, EDC, HOBt, DIPEA, THF, rt, overnight, 50 %; xiii) **16**, LiOH 1 M, THF, 2 h, 0 °C to rt, 74 %; xiv) **17**, HCl 4 M in dioxane, dioxane, 1 h, rt, 97 %.

Sodium (phenylamino)methanesulfonate [2]

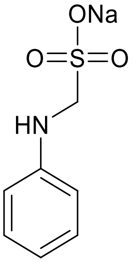

2

Compound **2** was synthesized according to literature[1] .

^{1}H NMR (400 MHz, D_2O) δ 7.29 (t, 2H), 6.92 (d, *J* = 7.7 Hz, 2H), 6.86 (t, *J* = 6.9 Hz, 1H), 4.44 (s, 2H).

3-azidopropan-1-amine [4]

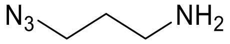

4

Compound **4** was synthesized according to literature[2] .

^{1}H NMR (400 MHz, $CDCl_3$) δ 3.35 (t, *J* = 6.7 Hz, 2H), 2.78 (t, *J* = 6.8 Hz, 2H), 1.71 (p, *J* = 6.8 Hz, 2H), 1.26 (s, 2H).

4-((3*R*,5*S*)-1-(*tert*-butoxycarbonyl)-5-(ethoxycarbonyl)-2-oxopyrrolidin-3-yl)butanoic acid [15]

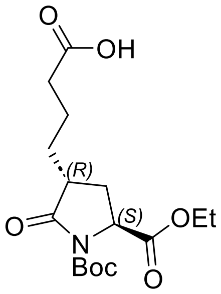

15

Pyroglutamate **15** was synthesized according to literature[4].

^{1}H NMR (400 MHz, $CDCl_3$) δ 4.55 (d, *J* = 9.6, 1.5 Hz, 1H), 4.23 (q, *J* = 7.1 Hz, 2H), 2.68 – 2.57 (m, 1H), 2.39 (m, 2H), 2.27 – 2.21 (m, 1H), 2.03 – 1.88 (m, 2H), 1.75 – 1.64 (m, 2H), 1.49 (s, 9H), 1.42 (m, 1H), 1.29 (t, *J* = 7.1 Hz, 3H).

(*E*)-4-((4-aminophenyl)diazenyl)-3-fluorobenzoic acid [8]

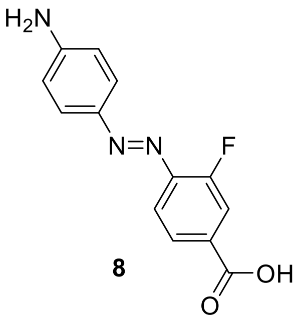

8

Compound **8** was synthesized following a reported procedure [3].

^{1}H-NMR and ^{13}C-NMR are in accordance with literature.

^{1}H NMR (400 MHz, DMSO) δ 7.82 (d, 2H), 7.74 – 7.68 (m, 3H), 6.69 (d, 2H), 6.45 (s, 1H).

^{13}C NMR (101 MHz, DMSO) δ 165.97 (d, $^{4}J_{C,F}$ = 2.7 Hz), 157.73 (d, $^{1}J_{C,F}$ = 253.4 Hz), 154.26, 143.41, 143.27 (d, $^{2}J_{C,F}$ = 7.3 Hz), 132.15 (d, $^{3}J_{C,F}$ = 6.9 Hz), 126.32, 125.84 (d, $^{4}J_{C,F}$ = 3.5 Hz), 117.62, 117.56 (d, $^{2}J_{C,F}$= 20.9 Hz), 113.51.

HPLC-MS: Rt: 2.76; $[M+H]^{+}$= 260

(*E*)-4-((4-aminophenyl)diazenyl)-N-(3-azidopropyl)-3-fluorobenzamide [9]

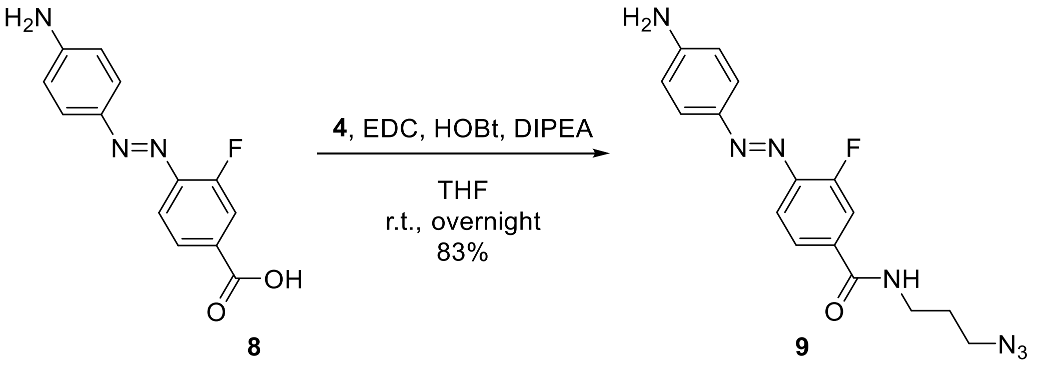

To a stirring solution of acid **8** (570 mg, 2.20 mmol), HOBt (460 mg, 3.41 mmol) and EDC (514 mg, 3.17 mmol) in anhydrous THF (80 mL), a solution of 3-azidopropan-1-amine (**4**, 286 mg, 2.86 mmol) and DIPEA (1.22 g, 9.48 mmol, 1.65 mL) in anhydrous THF (30 mL) was added. The resulting reddish mixture was left stirring at room temperature overnight. The next day, the solvent was evaporated under *vacuum* and the crude was taken up in water and DCM. The aqueous phase was extracted with DCM (3X70 mL), the organic phases were collected, dried over $MgSO_4$, and evaporated. The crude was purified over direct phase column chromatography (DCM:MeOH 98:2) affording a yield of 92 % (689 mg, 2.02 mmol).

^{1}H NMR (400 MHz, DMSO) δ 8.66 (t, *J* = 5.6 Hz, 1H), 7.83 (dd, *J* = 11.8, 1.8 Hz, 1H), 7.77 – 7.65 (m, 4H), 6.69 (d, 2H), 6.38 (s, 2H), 3.43 (t, *J* = 6.8 Hz, 2H), 3.38 – 3.33 (m, 3H), 1.80 (p, *J* = 6.8 Hz, 2H).

^{13}C NMR (101 MHz, DMSO) δ 164.58 (d, $^4J_{C,F}$ = 1.5 Hz), 157.82 (d, $^1J_{C,F}$ = 253.0 Hz), 153,99, 143.34, 141.98 (d, $^2J_{C,F}$ = 7.3 Hz), 135.97 (d, $^3J_{C,F}$ = 7.0 Hz), 126.10, 123.72 (d, $^4J_{C,F}$ = 3.1 Hz), 117.28, 115.69 (d, $^2J_{C,F}$ = 21.5 Hz), 113.46, 48.51, 36.80, 28.29.

HPLC-MS: Rt 2.72; $[M+H]^+$= 342

(*E*)-4-((4-(2-aminoacetamido)phenyl)diazenyl)-N-(3-azidopropyl)-3-fluorobenzamide [11]

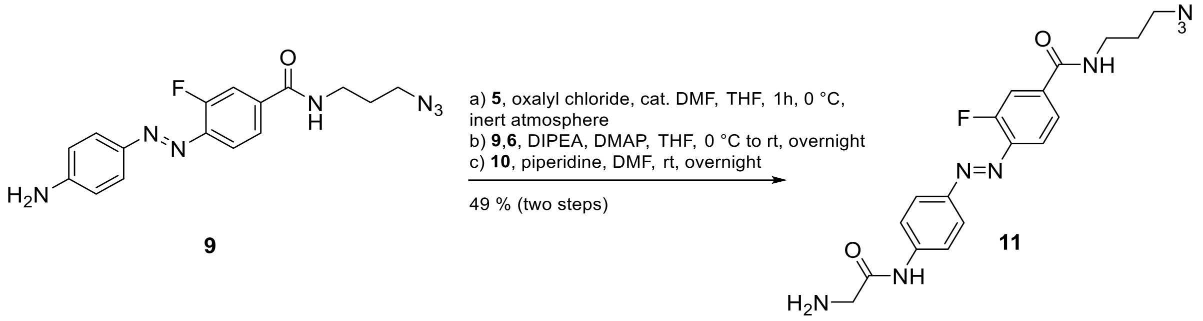

Compound **11** was synthesized following literature[5].

To a solution of Fmoc-Gly-OH (**5**, 1.09 g, 3.68 mmol) in THF (15 mL), oxalyl chloride was added (0.701 g, 5.52 mmol, 0.5 mL) and one drop of DMF and the mixture was stirred for 45 min at 0 °C. It was then allowed to cool to room temperature, diluted with toluene, and evaporated under reduced pressure.

The crude (1.16 g) was dissolved in anhydrous THF (17 mL) in an ice bath, under nitrogen. To this solution, a mixture of compound **9** (314 mg, 0.920 mmol), DIPEA (713 mg, 5.52 mmol, 0.962 mL) and DMAP (22 mg, 0.184 mmol) in anhydrous THF (17 mL) was added and the mixture was left stirring at 0° C for one hour and at room temperature overnight. The solvent was evaporated, and the crude was recrystallized from ethyl acetate.

The light orange crude (1.019 g) was dissolved in DMF (65 mL) and piperidine (783 mg, 9.20 mmol) was added and the resulting red solution was stirred overnight. The solvent was removed *under vacuum* and the crude was taken up in $NaHCO_3$ (100 mL) and EtOAc (100 mL). The aqueous phase was extracted with EtOAc (100 mL). The OPs were collected and washed with water and brine, dried over $MgSO_4$ and evaporated at the rotary evaporator.

The crude was purified over reverse phase column chromatography (H_2O/ACN gradient) affording compound **11** (178 mg, 0.447 mmol) as an orange powder (two steps, 49 % yield).

^{1}H NMR (400 MHz, CD_3OD) δ 7.96 (d, J = 8.8 Hz, 2H), 7.85 – 7.73 (m, 5H), 3.50 (t, 4H), 3.44 (t, J = 6.6 Hz, 2H), 1.90 (p, J = 6.8 Hz, 2H).

^{13}C NMR (101 MHz, DMSO) δ 164.44, 157.24, 147.84, 142.87, 141.36 (d, $^{2}J_{C,F}$ = 6.9 Hz), 137.97 (d, $^{3}J_{C,F}$ = 6.5 Hz), 124.37, 123.94 (d, $^{4}J_{C,F}$ = 3.3 Hz), 119.30, 117.61, 116.04 (d, $^{2}J_{C,F}$ = 21.5 Hz), 113.50, 54.91, 48.52, 36.88, 28.28.

HPLC-MS: Rt: 1.72; $[M+H]^+$= 399

1-(*tert*-butyl) 2-ethyl (2*S*,4*R*)-4-(4-((2-((4-((*E*)-(4-((3-azidopropyl)carbamoyl)-2-fluorophenyl)diazenyl)phenyl)amino)-2-oxoethyl)amino)-4-oxobutyl)-5-oxopyrrolidine-1,2-dicarboxylate [16]

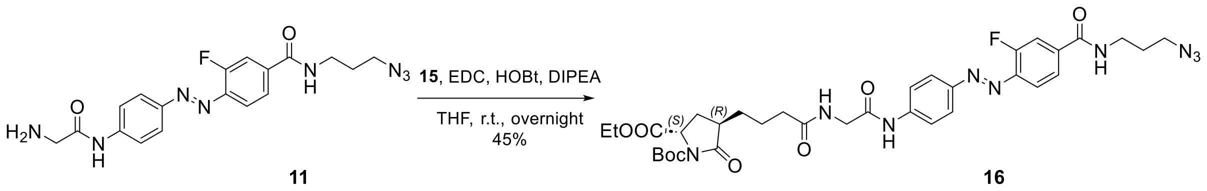

To a stirred solution of compound **11** (135 mg, 0.339 mmol) in THF (18 mL), a solution of **15** (128 mg, 0.373 mmol), HOBT (70.06 mg, 0.518 mmol), EDC (86.40 mg, 0.451 mmol) and DIPEA (179 mg, 1.39 mmol, 0.242 mL) in anhydrous THF (12 mL) was added and the reaction was stirred under N_2 overnight.

The following day, 50 mL of water was added to the mixture, and the aqueous phase was extracted with DCM (3 × 20 mL). The organic phases were combined, dried over $MgSO_4$, and evaporated under reduced pressure. The crude (reddish powder) was purified over reverse phase column chromatography (H_2O/ACN gradient) affording compound **16** (121 mg, 0.168 mmol) as an orange powder (50 % yield).

^{1}H NMR (400 MHz, CD_3OD) δ 7.95 (d, *J* = 9.0 Hz, 2H), 7.83 – 7.71 (m, 5H), 4.67 – 4.61 (m, 1H), 4.30 – 4.20 (m, 2H), 4.04 (s, 2H), 3.49 (t, *J* = 6.9 Hz, 2H), 3.43 (t, *J* = 6.7 Hz, 2H), 2.65 (ddt, *J* = 13.5, 9.0, 4.8 Hz, 1H), 2.35 (td, *J* = 7.3, 2.1 Hz, 2H), 2.32 – 2.25 (m, 1H), 2.15 – 2.03 (m, 1H), 1.95 – 1.82 (m, 4H), 1.72 (p, *J* = 7.4 Hz, 2H), 1.48 (s, 9H), 1.30 (td, *J* = 7.1, 1.7 Hz, 3H).

^{13}C NMR (101 MHz, CD_3OD) δ 177.81, 176.34, 173.11, 169.97, 168.15, 162.00, 159.45, 150.87, 150.39, 143.66, 139.23, 125.45, 124.52, 121.05, 118.93, 117.22, 84.72, 62.88, 58.85, 50.23, 44.16, 42.67, 38.61, 36.43, 30.95, 29.72, 29.08, 28.11, 23.95, 14.53.

HPLC-MS: Rt: 2.98; $[M+Na]^+$= 746, $[(M\text{-}Boc)+H]^+$= 624

(2*R*,4*S*)-2-(4-((2-((4-((*E*)-(4-((3-azidopropyl)carbamoyl)-2-fluorophenyl)diazenyl)phenyl)amino)-2-oxoethyl)amino)-4-oxobutyl)-4-((tert-butoxycarbonyl)amino)pentanedioic acid [17]

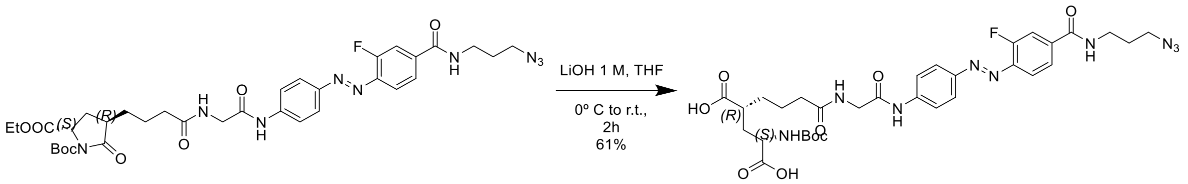

Compound **17** was synthesized as described in the literature.[6]

Compound **16** (77.8 mg, 0.107 mmol) was stirred in anhydrous THF (5 mL) in an ice-bath. LiOH 1 M (1.37 ml) was added dropwise, and the mixture turned reddish. The reaction was left stirring at 0 °C for two hours.

The solution was added of HCl 1 M until pH 4, and then it was added of MilliQ water (20 mL) and EtOAc (20 mL), keeping the temperature at 0 °C. The aqueous phase was separated off and extracted with EtOAc (3x20 mL). The organic phases were collected, dried over $MgSO_4$ and evaporated.

The crude (orange powder) was purified over reverse phase column chromatography (H_2O/ACN 0.1 % HCOOH gradient) affording compound **17** (54 mg, 0.076 mmol) as an orange powder (74 % yield).

^{1}H NMR (400 MHz, CD_3OD) δ 7.93 (d, J = 8.9 Hz, 2H), 7.82 – 7.69 (m, 5H), 4.16 – 4.09 (m, 1H), 4.05 (d, J = 3.8 Hz, 2H), 3.48 (t, J = 6.9 Hz, 2H), 3.43 (t, J = 6.6 Hz, 2H), 2.62 – 2.52 (m, 1H), 2.34 (t, J = 6.8 Hz, 2H), 2.26 – 2.16 (m, 1H), 1.90 (p, J = 6.8 Hz, 2H), 1.79 – 1.64 (m, 4H), 1.64 – 1.56 (m, 1H), 1.43 (s, 9H).

^{13}C NMR (101 MHz, CD_3OD) δ 178.70, 176.37, 176.08, 169.98, 168.10, 161.97, 159.42, 158.16, 150.34, 143.58, 139.11, 125.44, 124.48, 121.04, 118.90, 117.20, 80.50, 53.55, 50.22, 44.13, 43.01, 38.61, 36.41, 34.88, 33.32, 29.70, 28.73, 24.35.

HPLC-MS: Rt: 2.58; $[M+Na]^+$= 736, $[(M-Boc)+H]^+$= 614

(1*S*,3*R*)-7-((2-((4-((*E*)-(4-((3-azidopropyl)carbamoyl)-2-fluorophenyl)diazenyl)phenyl)amino)-2-oxoethyl)amino)-1,3-dicarboxy-7-oxoheptan-1-aminium chloride [18]

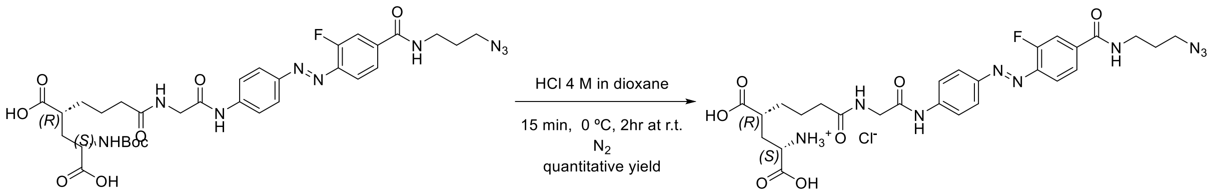

17 **18**

In a nitrogen purged flask, compound **17** (9.8 mg, 0.060 mmol) was dissolved in dioxane (2 mL) and added of 1 mL of 4 M HCl in dioxane. The suspension was stirred at 0 °C for 15 minutes, then at room temperature for 2 hours. Nitrogen gas was passed through the mixture to remove HCl, and it was then dried completely under reduced pressure. The crude red oil was triturated with diethyl ether, hexane, and ethyl acetate.

The final red powder was dried under vacuum. (8.7 mg 0.060 mmol, 97% yield).

^{1}H NMR (400 MHz, CD_3OD) δ 7.96 (d, *J* = 8.9 Hz, 2H), 7.84 – 7.71 (m, 5H), 4.06 (s, 2H), 4.03 – 3.96 (m, 1H), 3.49 (t, *J* = 6.8 Hz, 2H), 3.43 (t, *J* = 6.7 Hz, 2H), 2.75 – 2.64 (m, 1H), 2.42 – 2.31 (m, 3H), 1.97 – 1.86 (m, 3H), 1.84 – 1.66 (m, 4H).

^{13}C NMR (101 MHz, CD_3OD) δ 207.63, 177.81, 176.35, 170.01, 168.10, 161.99, 159.43, 150.40, 143.61, 139.16, 125.44, 124.50, 121.06, 118.91, 117.22, 62.18, 50.23, 44.10, 42.29, 38.62, 36.24, 33.28, 32.45, 29.71, 23.86.

HPLC-MS: Rt: 2.00; $[M+H]^+$= 614

Supporting Figures

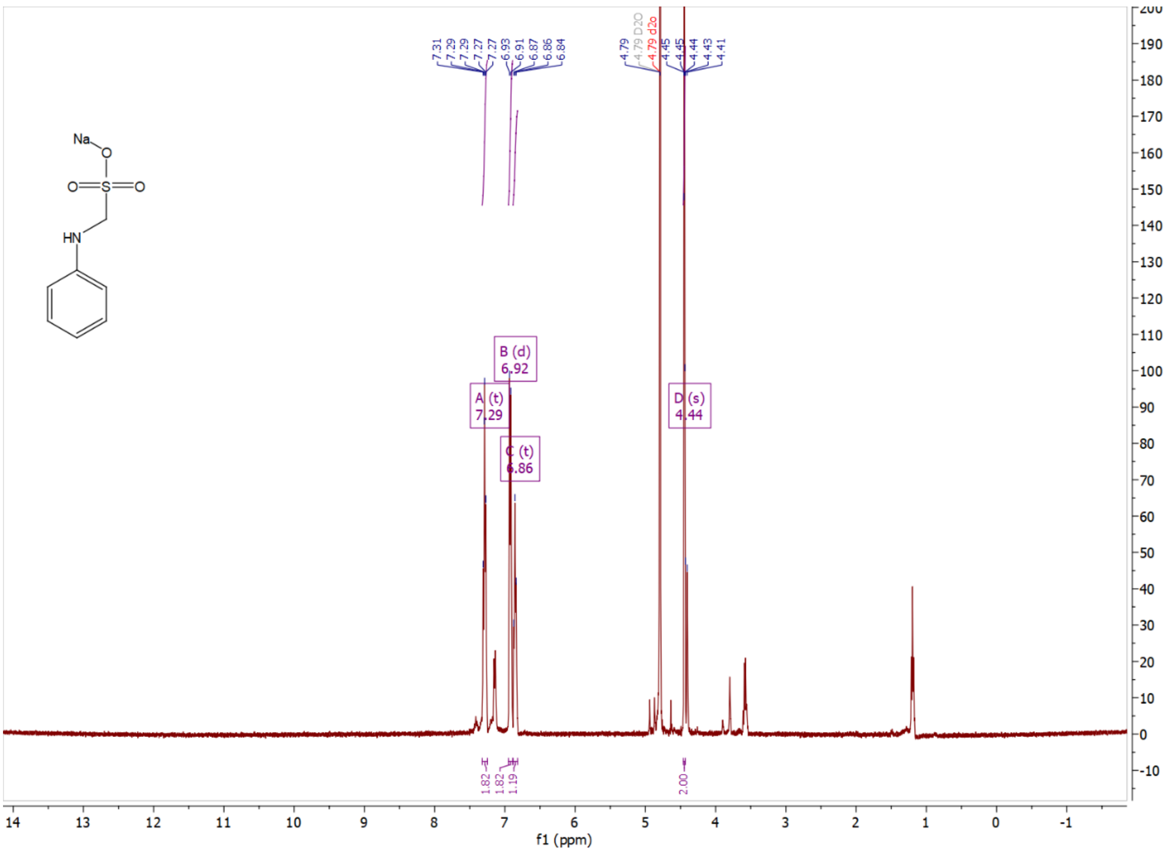

Figure S1. ^{1}H-NMR spectrum of compound **2**.

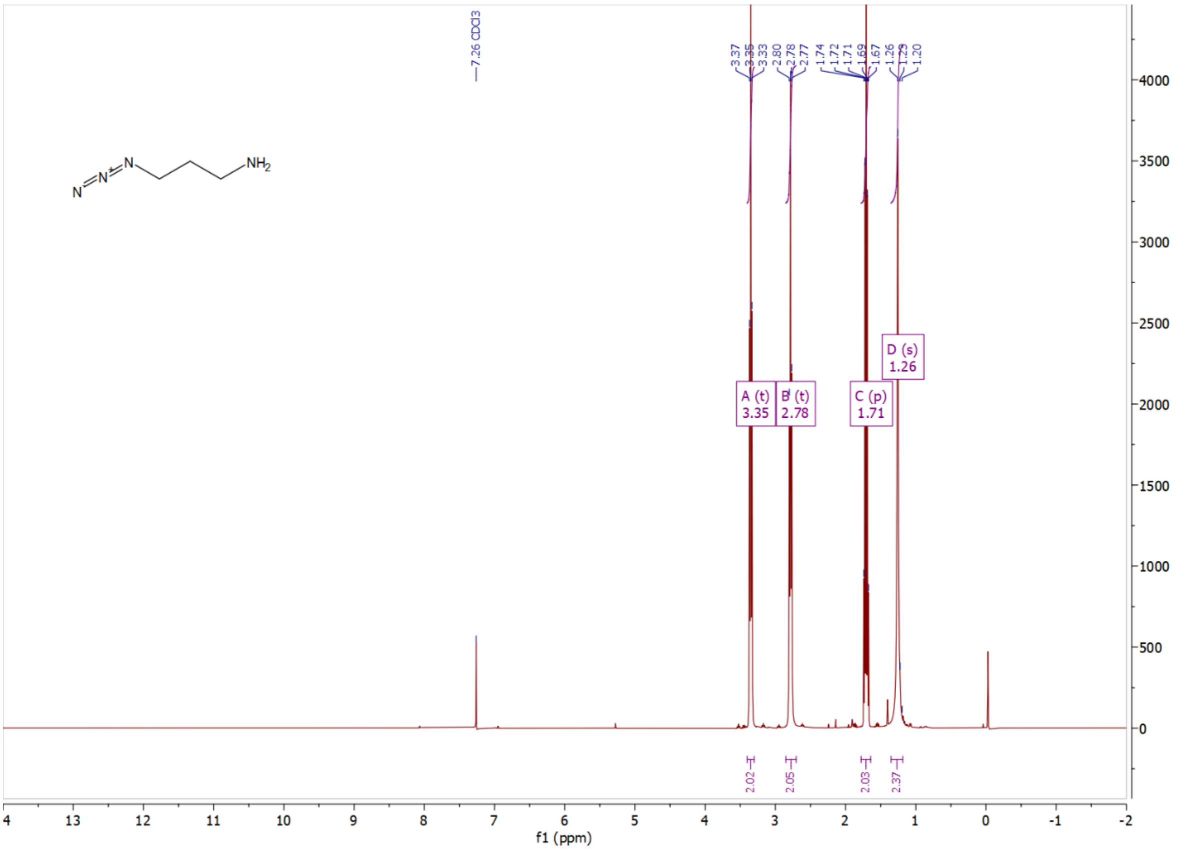

Figure S2. ^{1}H-NMR spectrum of compound **4**.

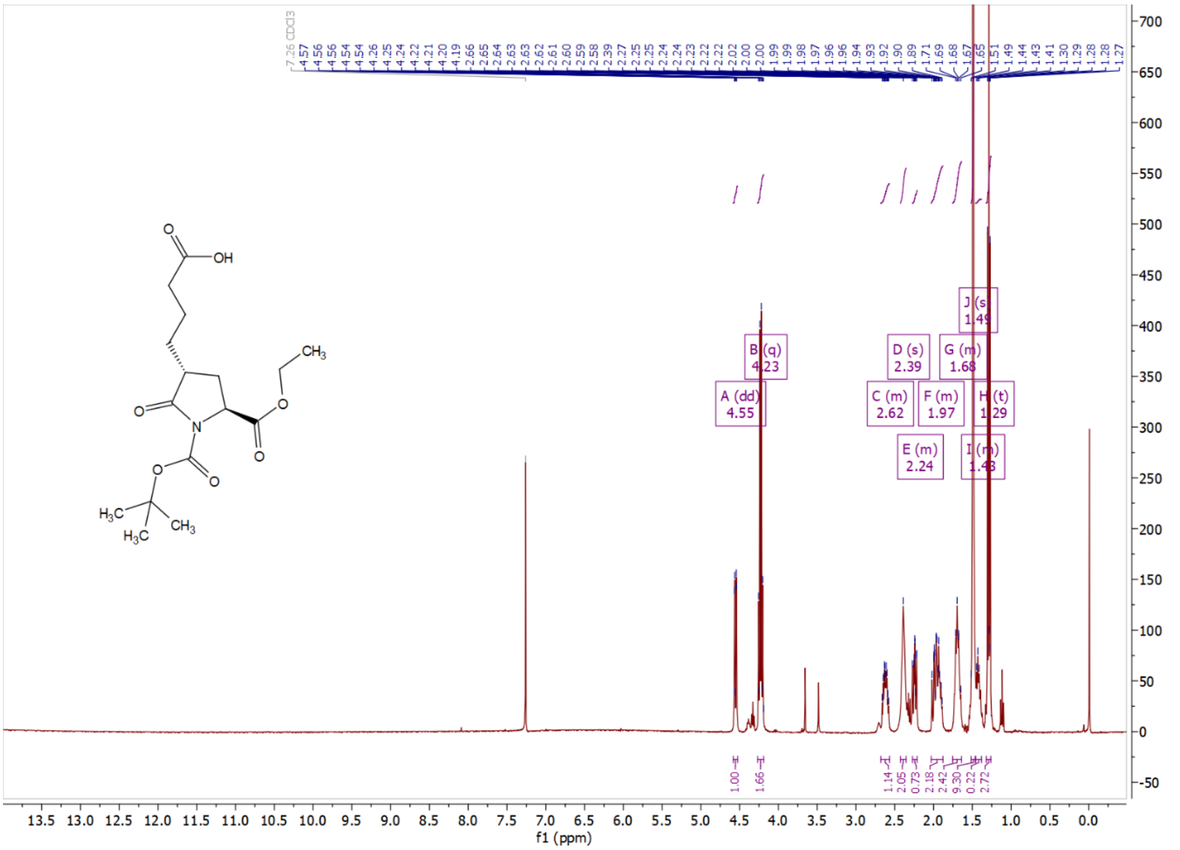

Figure S3. ^{1}H-NMR spectrum of compound **15**.

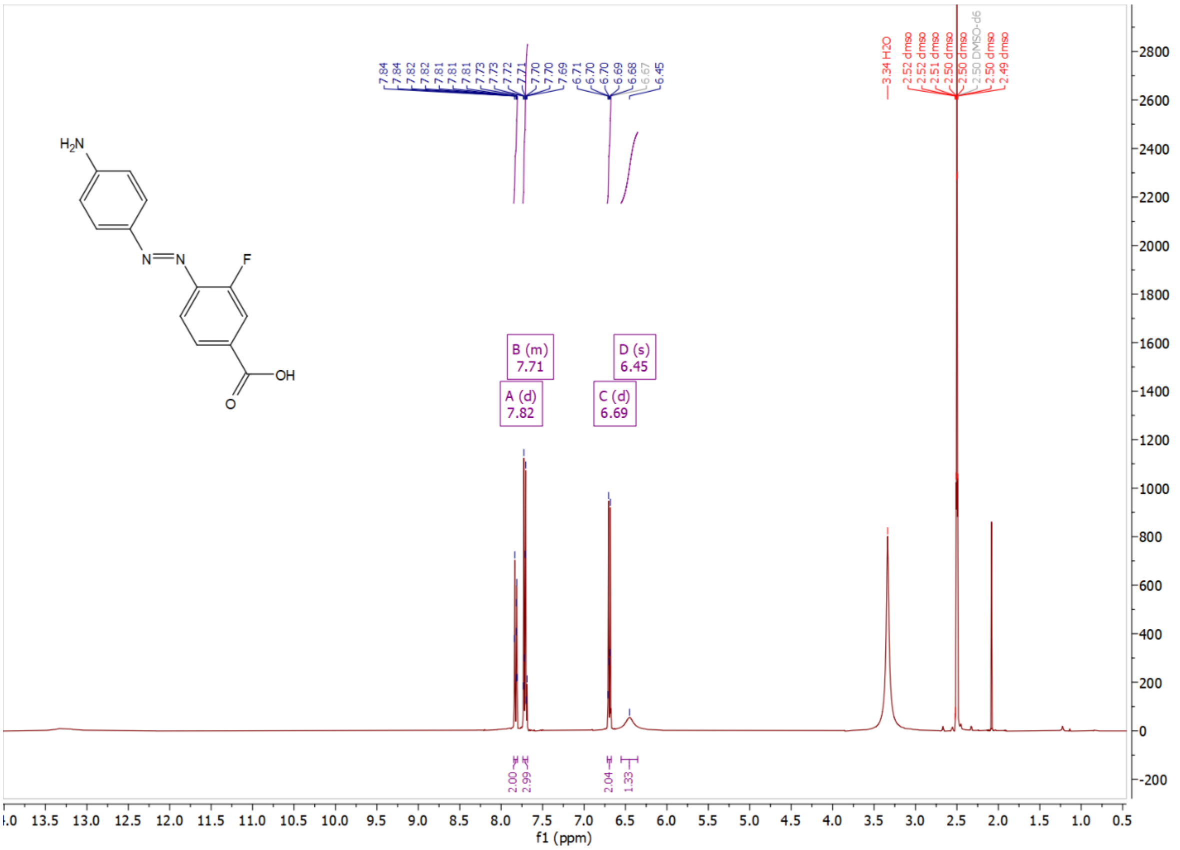

Figure S4. ^{1}H-NMR spectrum of compound **8**.

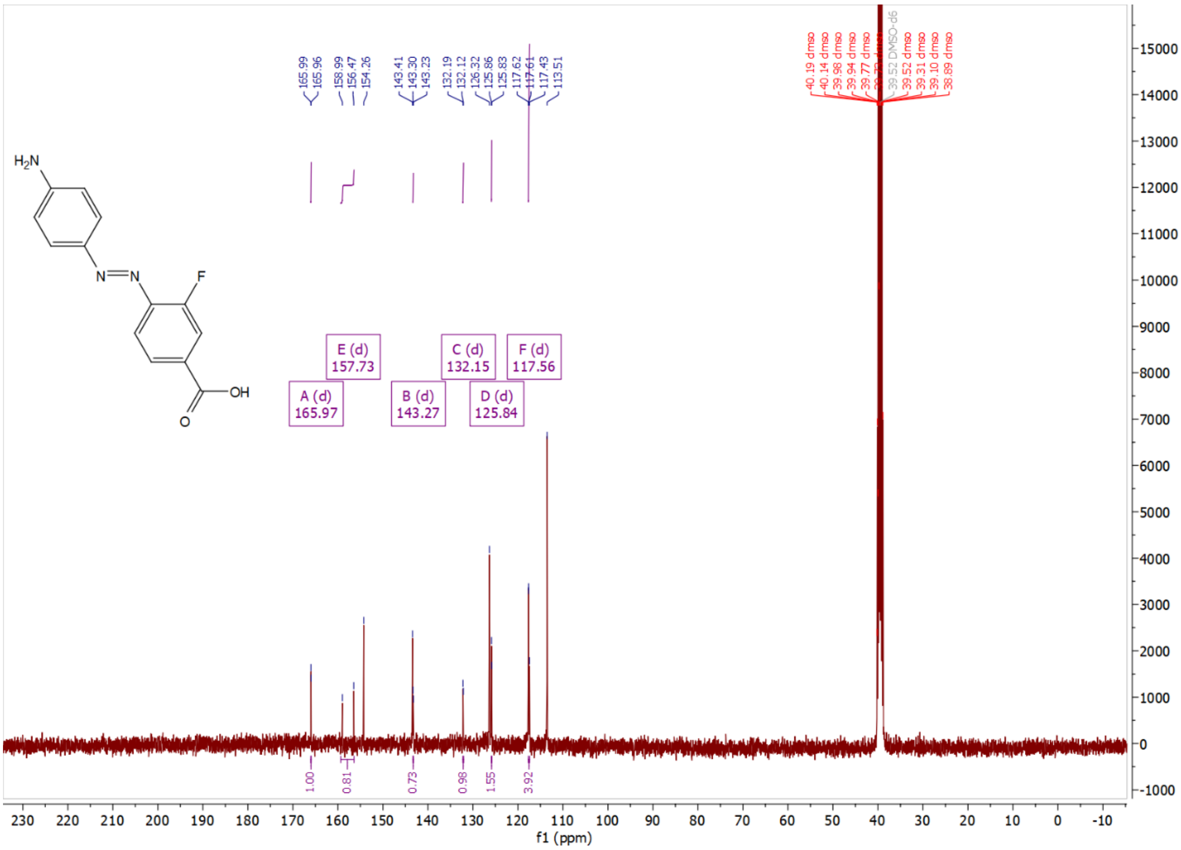

Figure S5. ^{13}C-NMR spectrum of compound **8**.

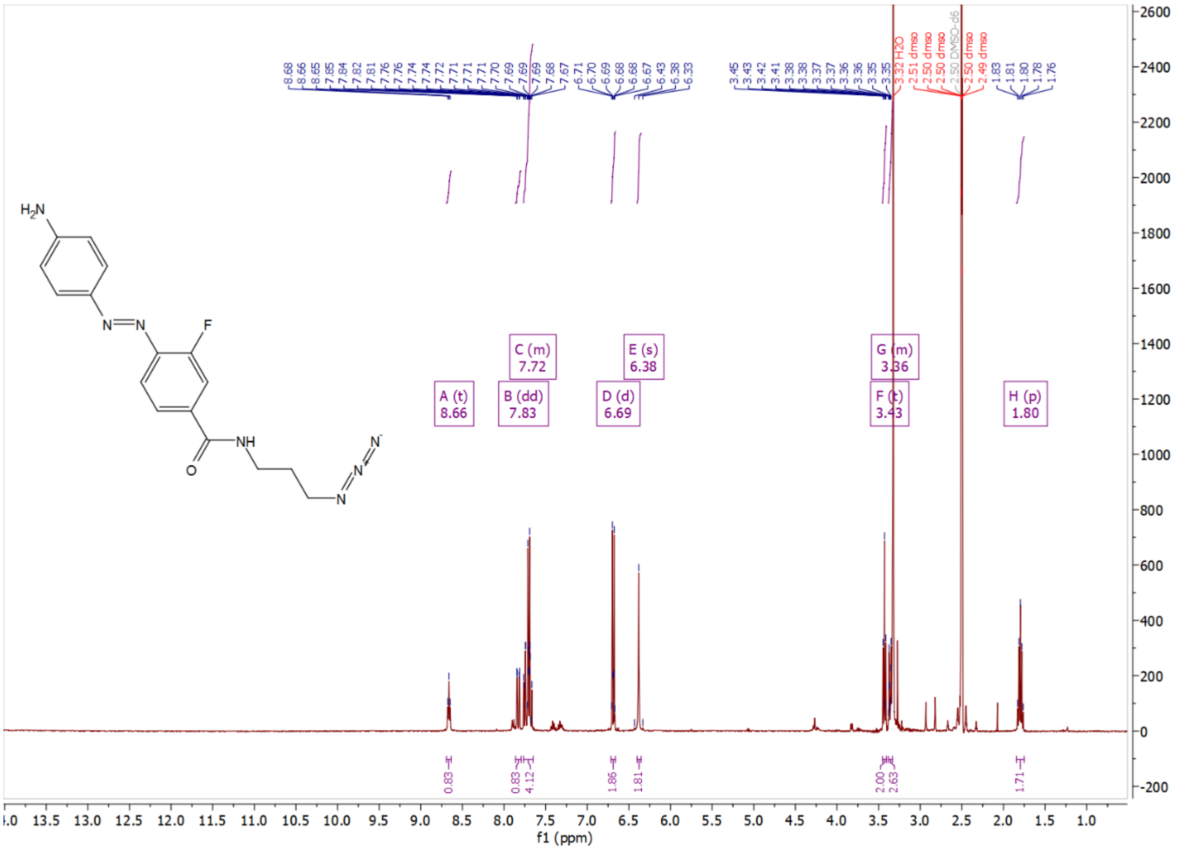

Figure S6. ^{1}H-NMR spectrum of compound **9**. See **Figure S16** for magnification of the aromatic region.

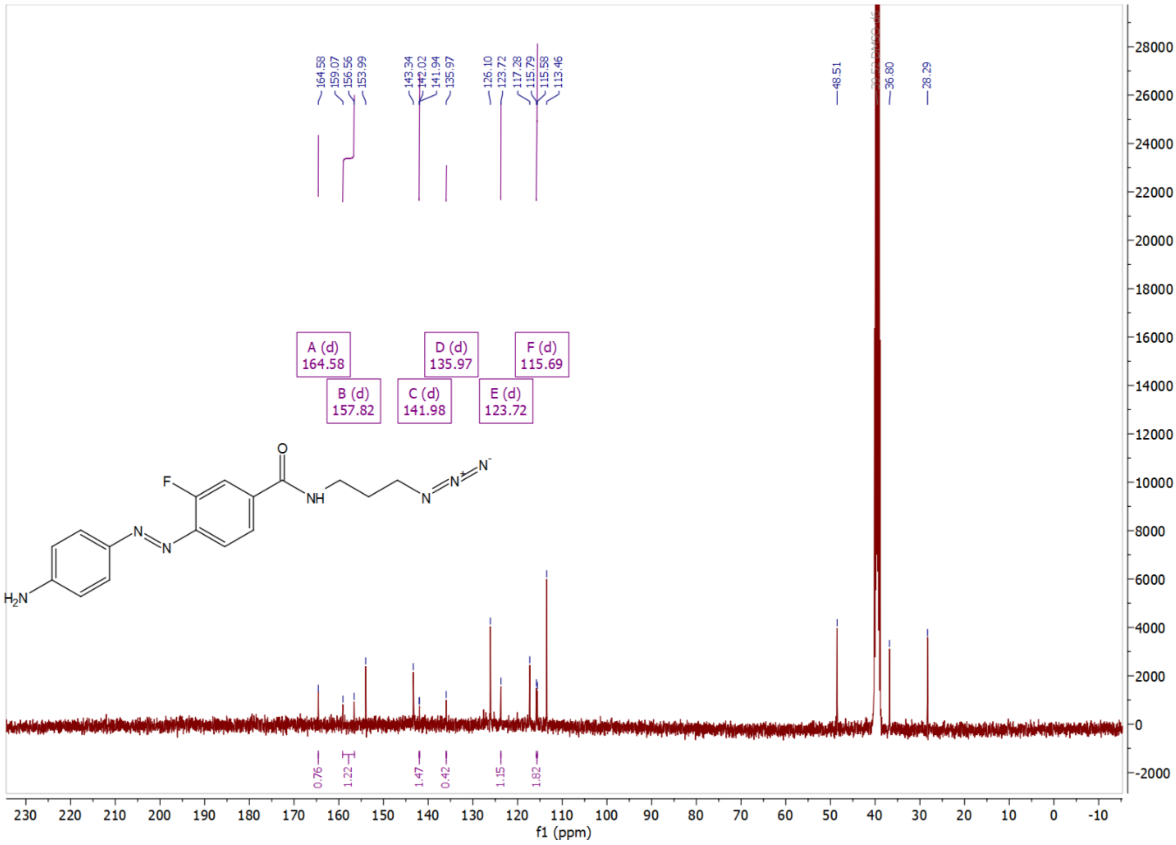

Figure S7. ^{13}C-NMR spectrum of compound **9**.

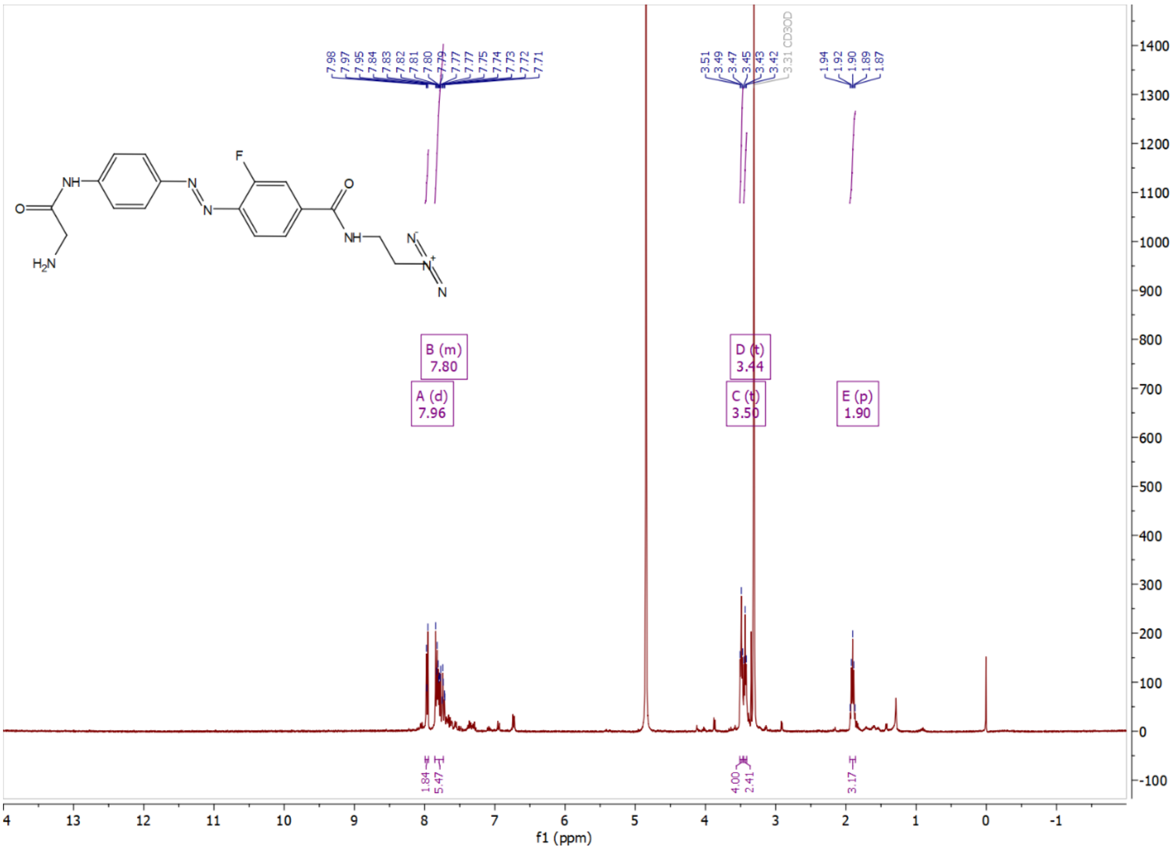

Figure S8. ^{1}H-NMR spectrum of compound **11**. See **Figure S16** for magnification of the aromatic region.

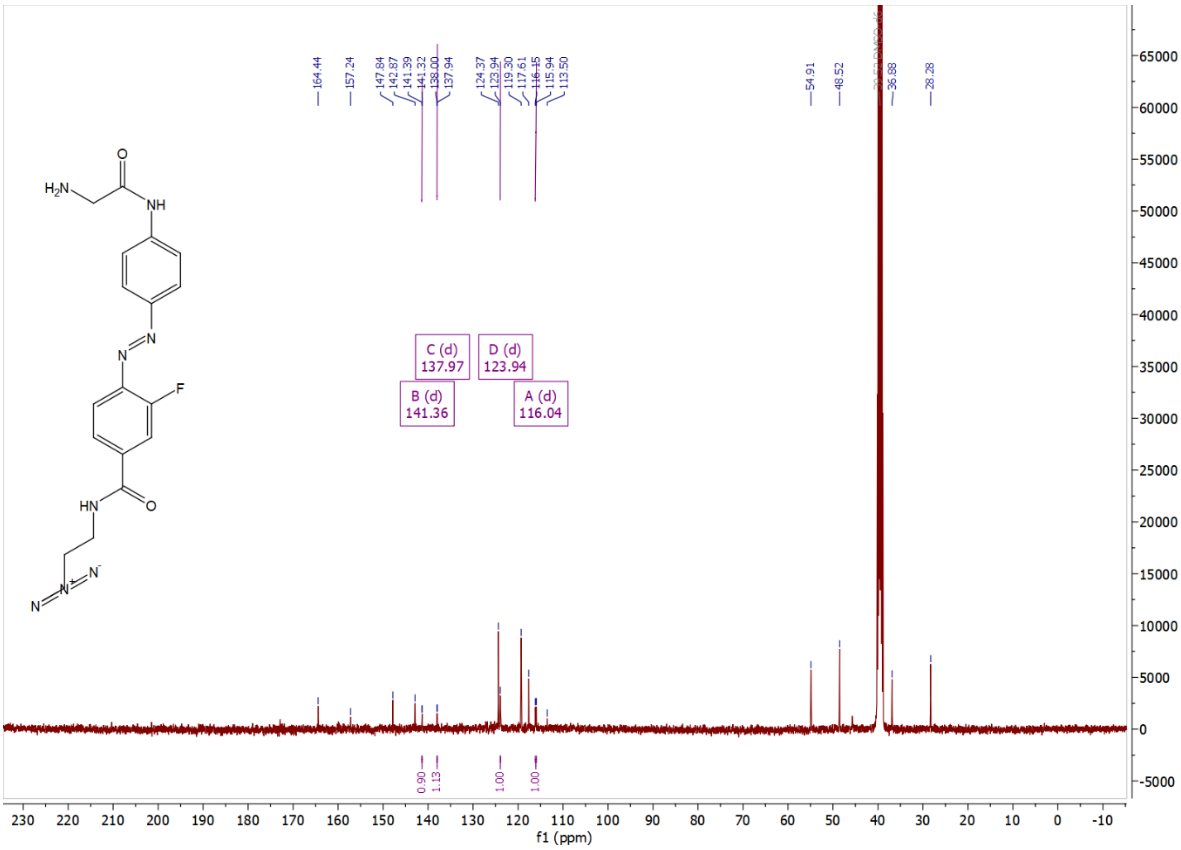

Figure S9. ^{13}C-NMR spectrum of compound **11**.

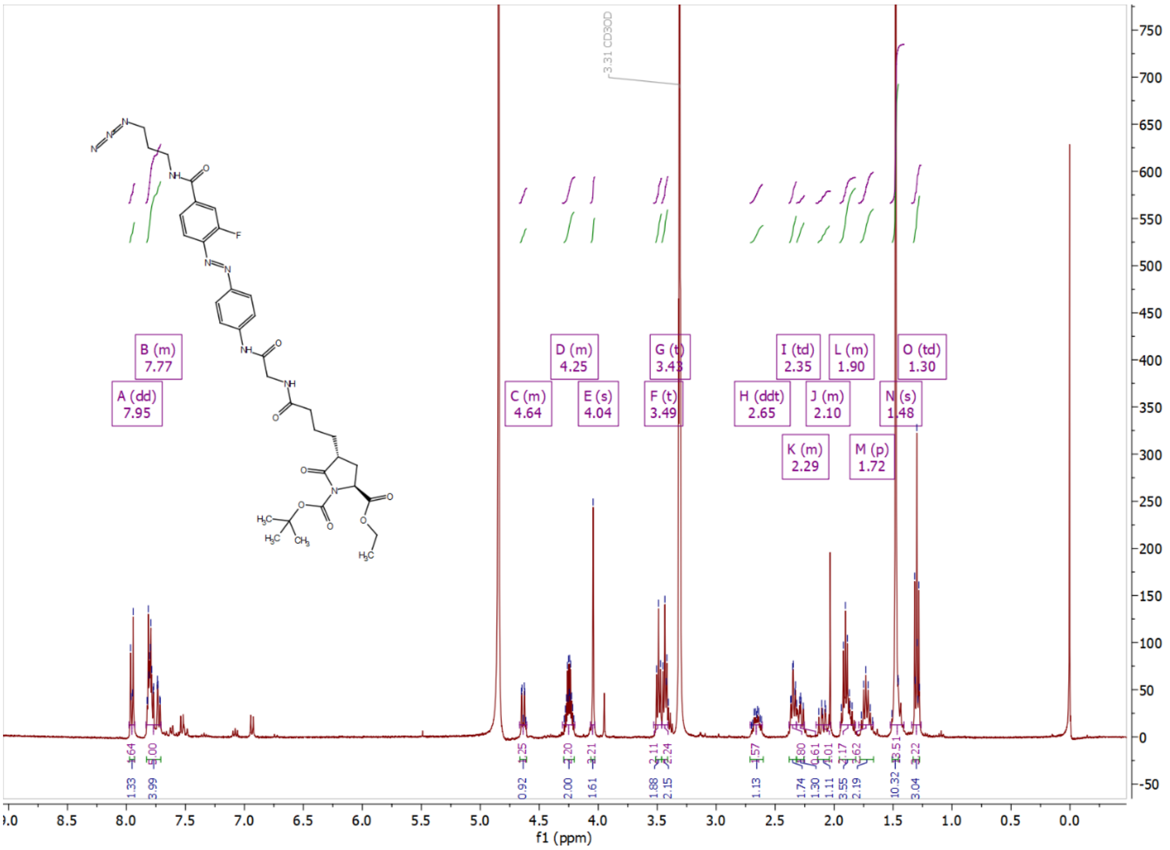

Figure S10. ^{1}H-NMR spectrum of compound **16**. See **Figure S16** for magnification of the aromatic region.

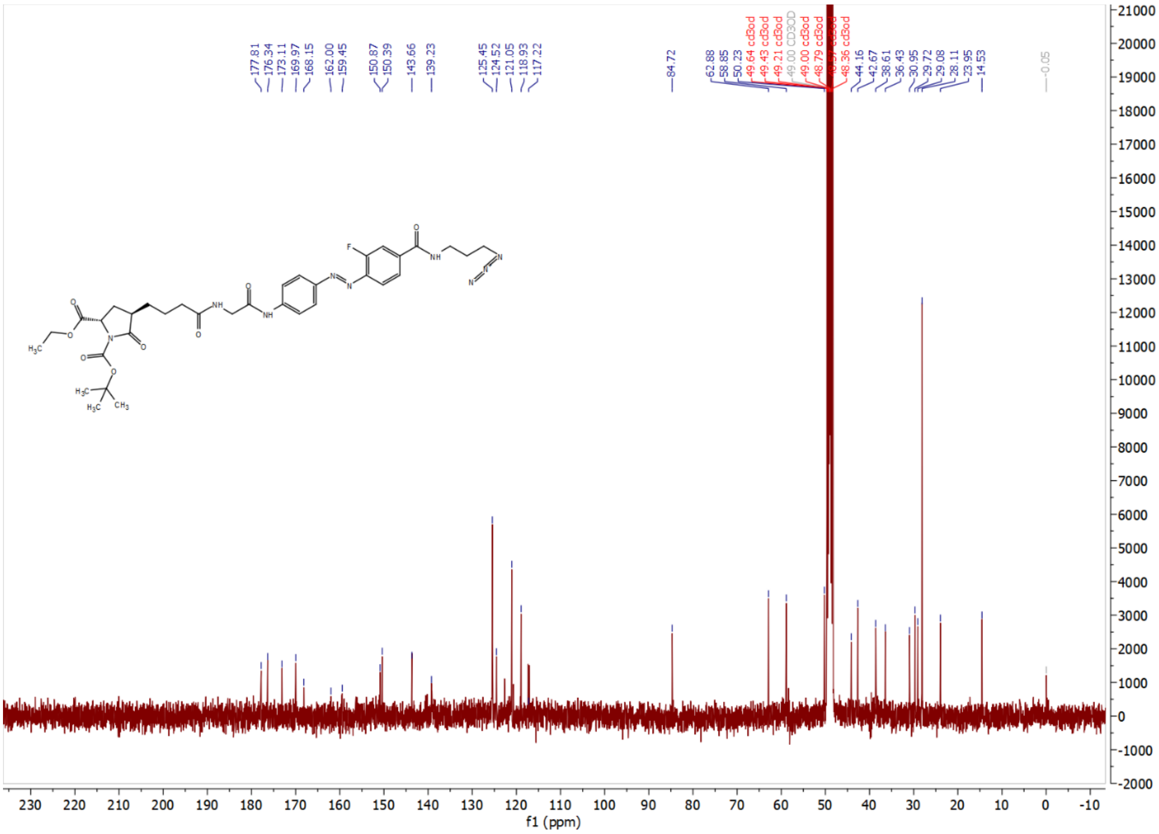

Figure S11. ^{13}C-NMR spectrum of compound **16**.

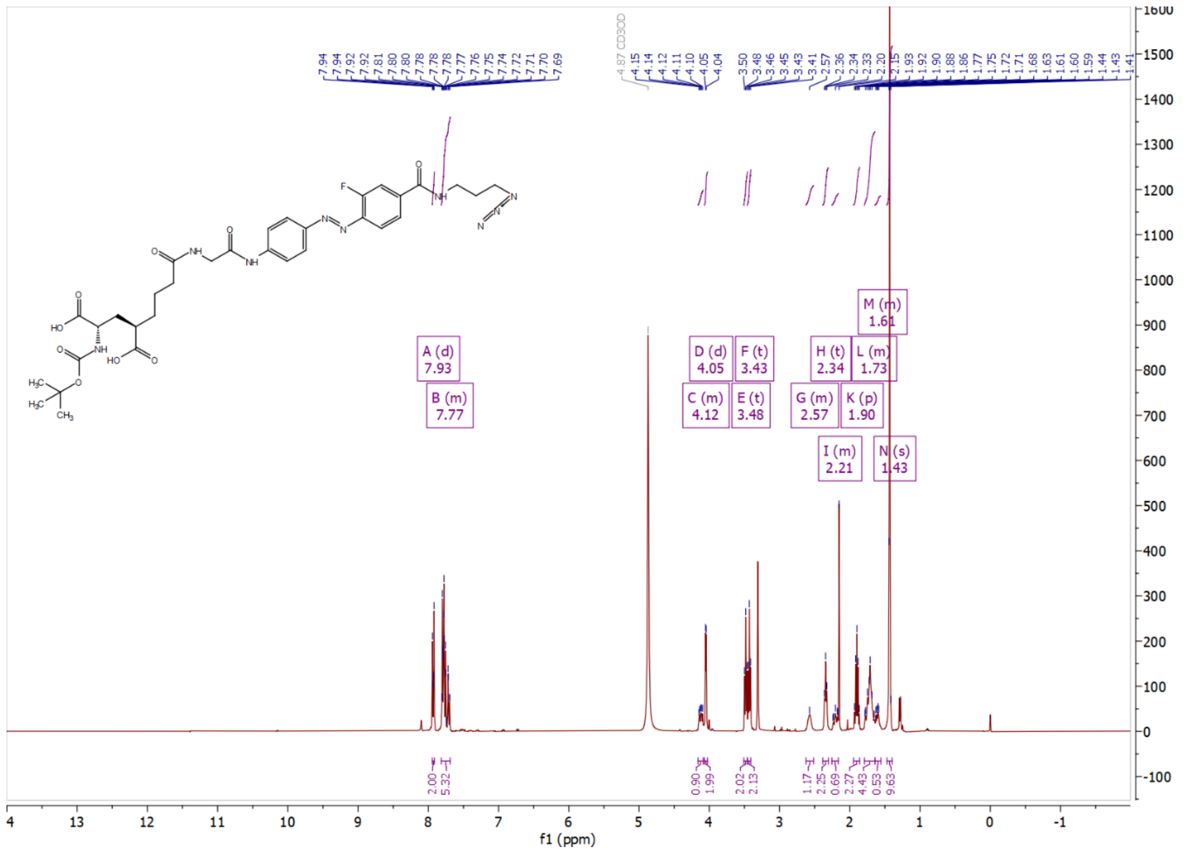

Figure S12. ^{1}H-NMR spectrum of compound **17**.

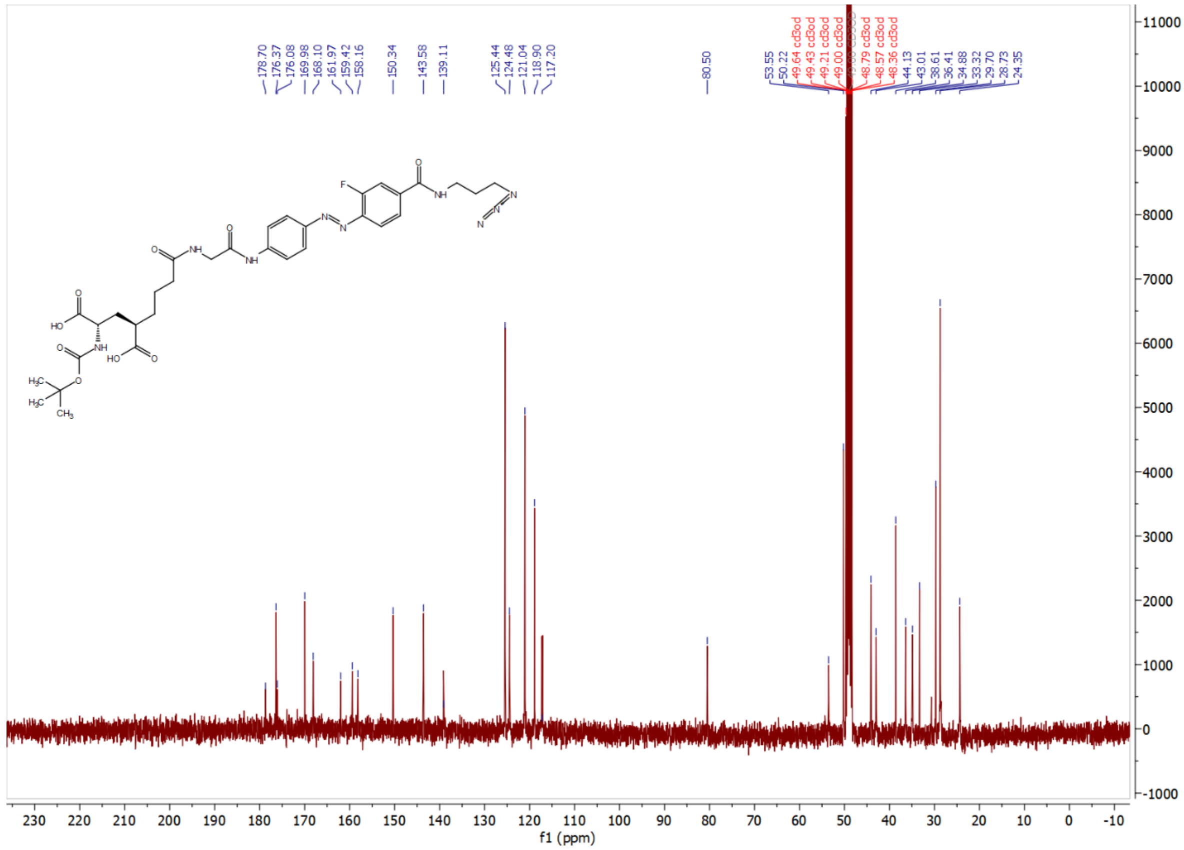

Figure S13. ^{13}C-NMR spectrum of compound **17**.

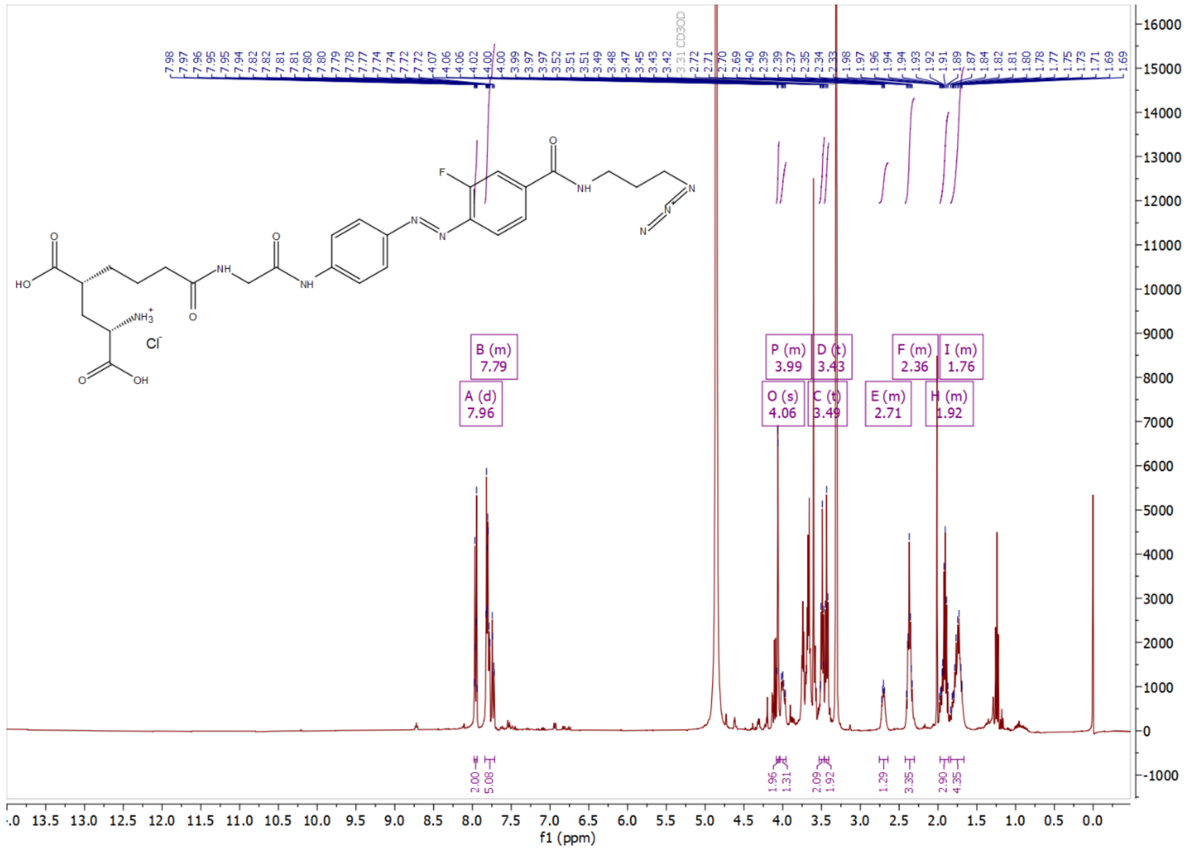

Figure S14. ^{1}H-NMR spectrum of compound **18**. See **Figure S16** for magnification of the aromatic region.

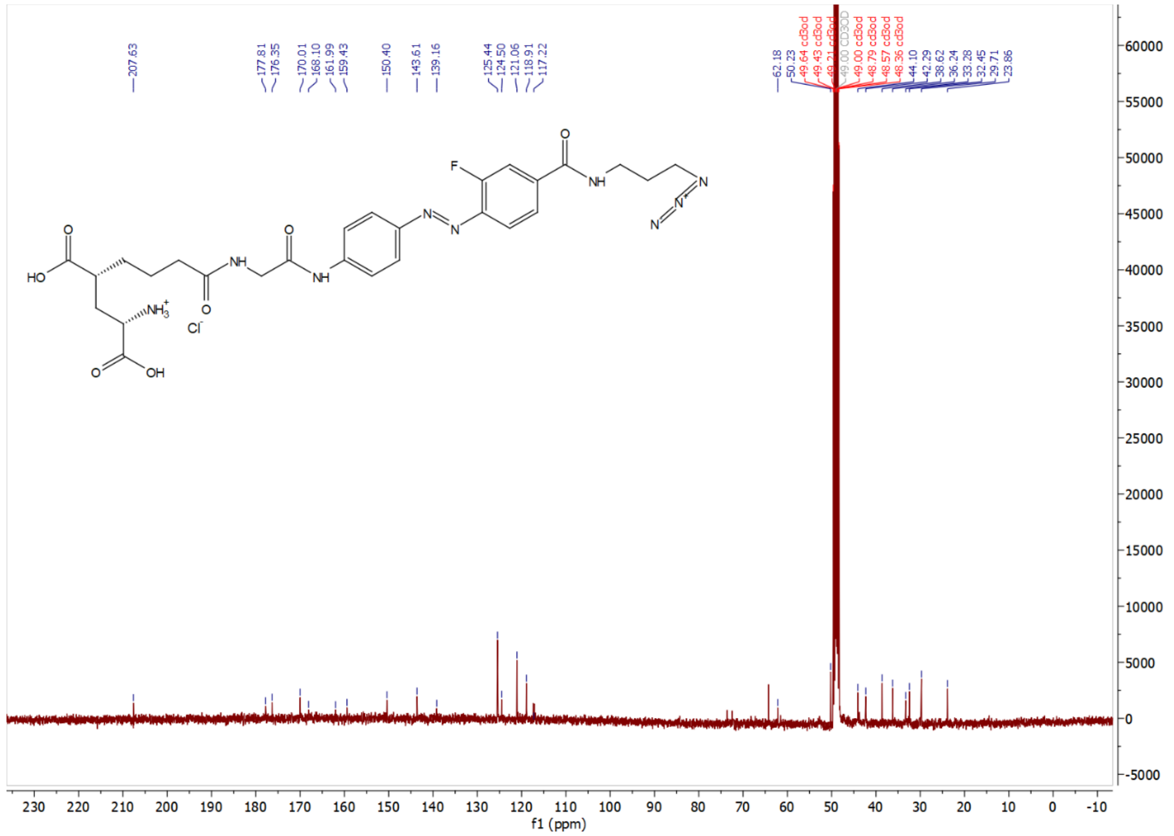

Figure S15. ^{13}C-NMR spectrum of compound **18**

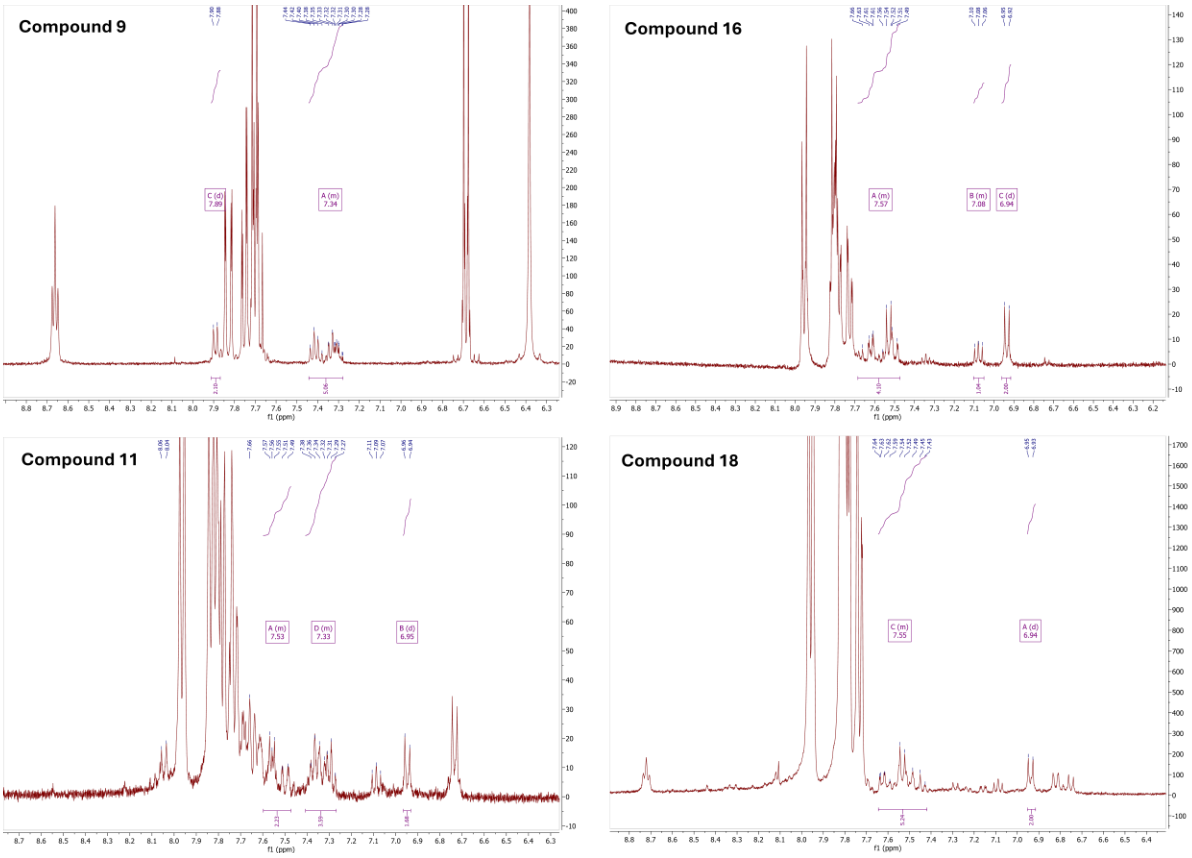

Figure S16. ^{1}H-NMR spectrum magnification of the aromatic region of compounds **9**, **11**, **16** and **18.** The presence of an equilibrium between *trans* and *cis* isomers can be observed in the aromatic region. In the case of intermediate **9**, peaks at 7.34 ppm and 7.89 ppm are representative of the *cis* isomer, while other peaks are traces of impurities and residual solvents (e.g. 2.07 ppm, acetonitrile in Figure S6). Intermediate **11** shows peaks between 6.91 ppm and 8.06 ppm that represents the *cis* isomer, while others are impurities (e.g. 6.75 ppm, compound **9**). Intermediate **16** shows peaks between 6.92 ppm and 7.96 ppm that are relative to the *cis* isomer. Intermediate **18** shows additional peaks between 6.71 and 8.73 ppm that are representative of the *cis* isomer and other side-products, possibly related to compound **9** (8.71 ppm and 6.75 ppm). Additional peaks between 3.56 ppm and 3.76 ppm (Figure S14) are related to impurities. The presence of *trans* and *cis* isomers, as well as traces of impurities related to compound **9** can be also appreciated by HPLC-PDA-MS, reported in following figures S17-21.

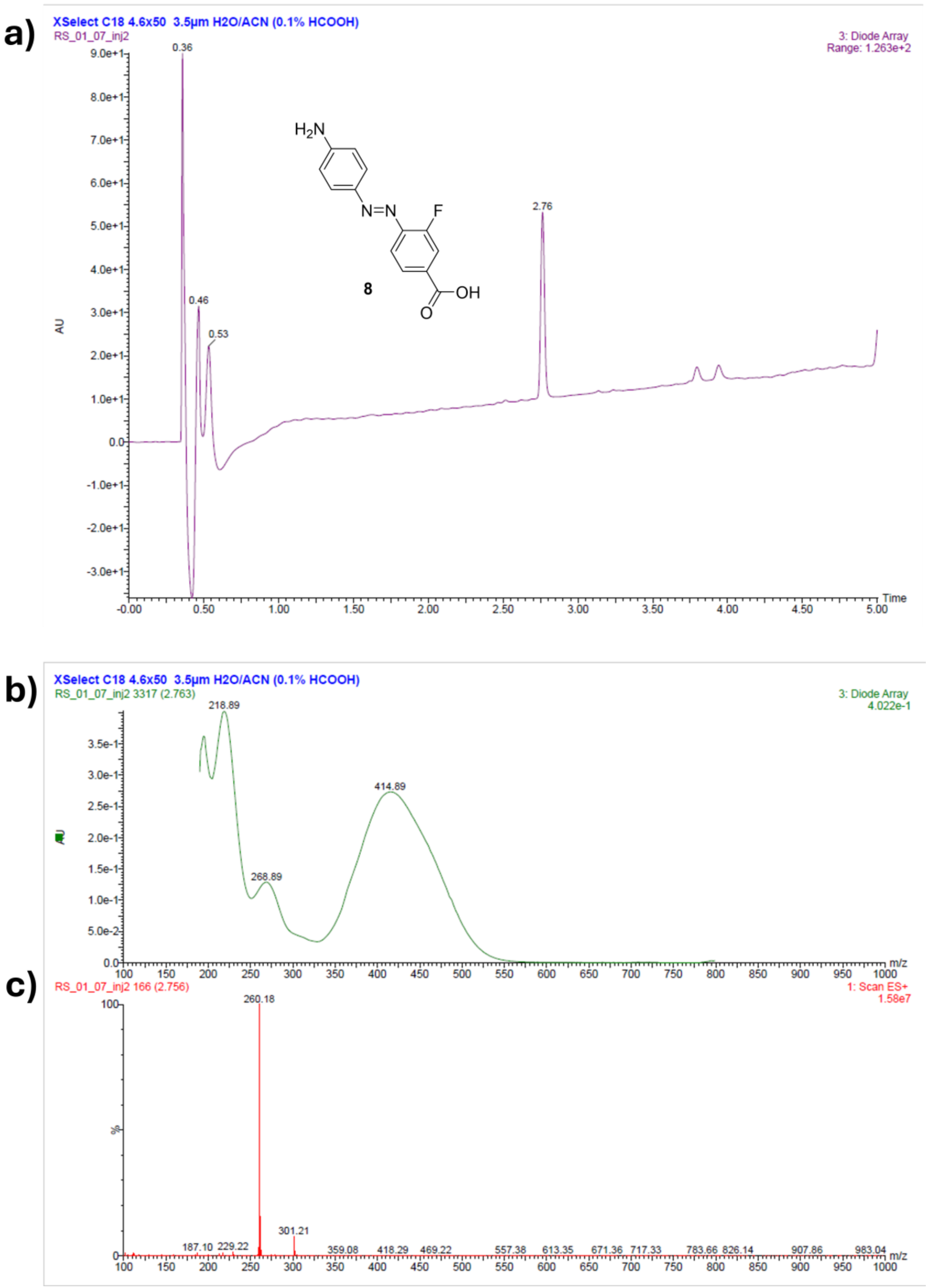

Figure S17. HPLC-PDA-MS of *trans* compound **8**. **a**) HPLC-PDA chromatogram of compound **8**. **b**) HPLC-PDA trace of *trans* compound **8** (Rt: 2.76 min; λ: 415 nm. **c**) HPLC-MS signal of compound **8** [$(M+H)^+$=260]. Panel b shows the characteristic absorption spectrum of the *trans* isomer of azobenzene.

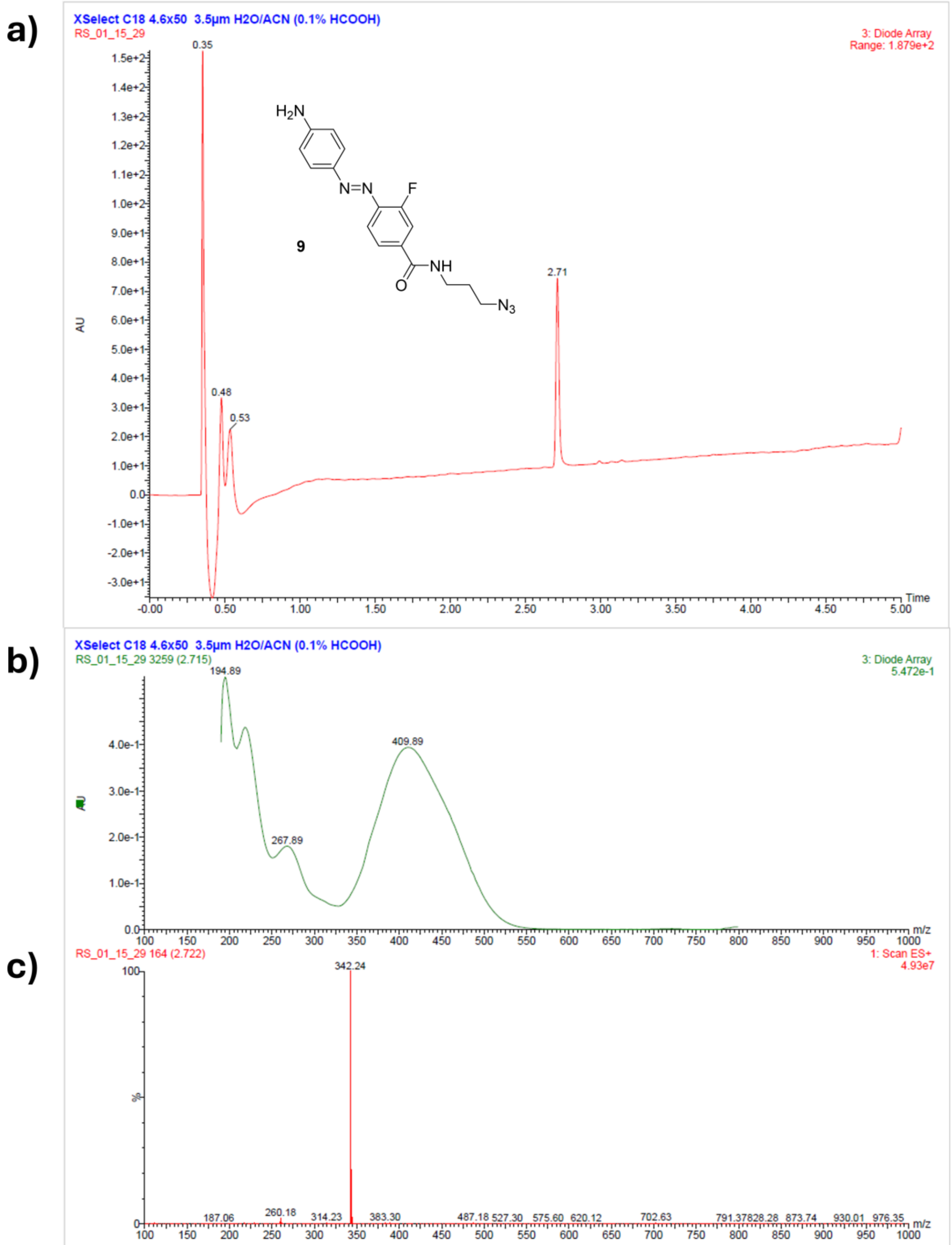

Figure S18. HPLC-PDA-MS of *trans* compound **9**. **a**) HPLC-PDA chromatogram of *trans* compound **9**. **b**) HPLC-PDA trace of *trans* compound **9** (Rt: 2.71 min; λ: 410 nm. **c**) HPLC-MS signal of compound **9** [(M+H)$^+$=342]. Panel b shows the characteristic absorption spectrum of the *trans* isomer of azobenzene.

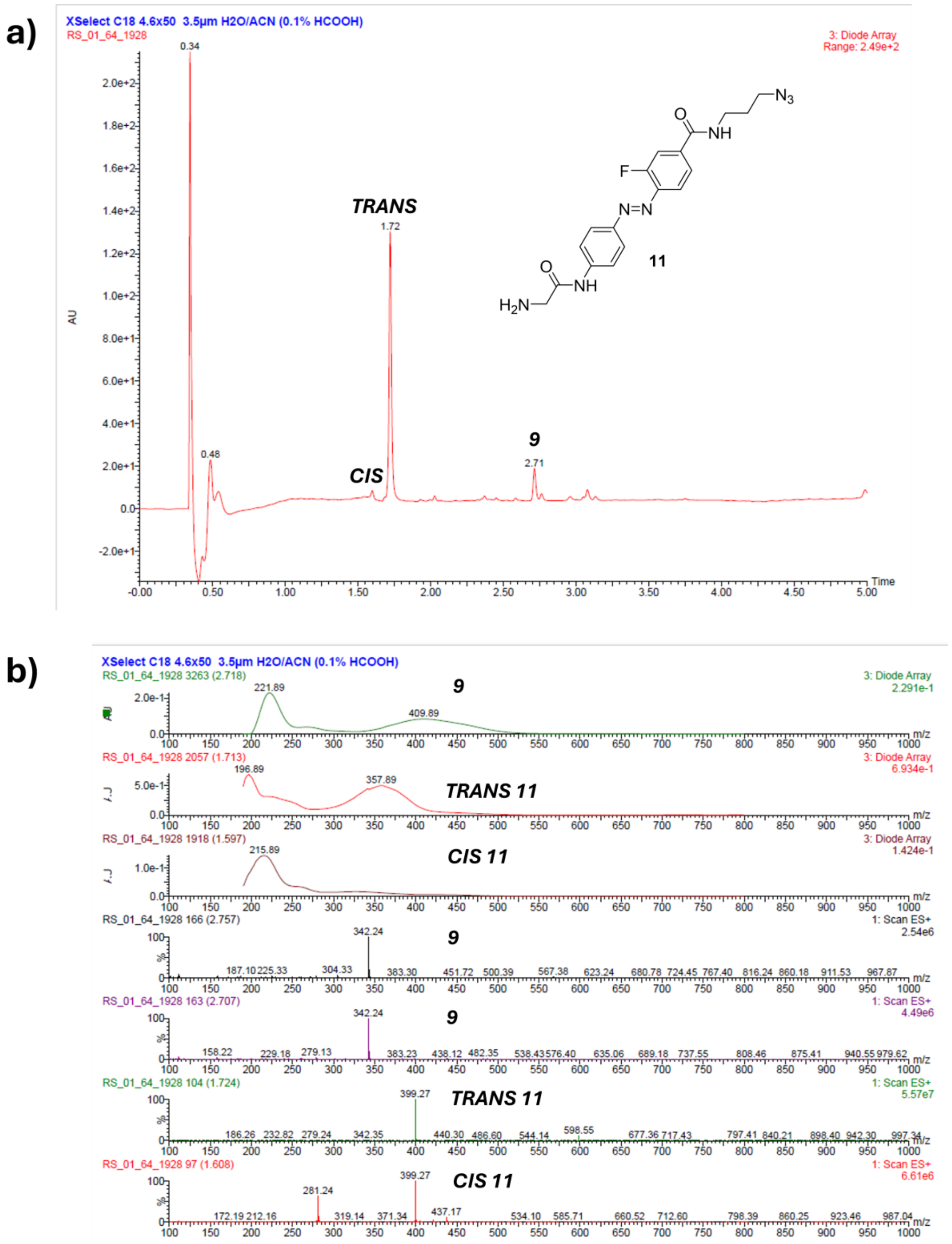

Figure S19. HPLC-PDA-MS of compound **11**. **a**) HPLC-PDA chromatogram of compound **11**. **b**) From top: HPLC-PDA trace of compound **9** (impurity, Rt: 2.71 min; λ: 410 nm); HPLC-PDA trace of *trans* compound **11** (Rt: 1.71 min; λ: 358 nm); HPLC-PDA trace of *cis* compound **11** (Rt: 1.60 min); HPLC-MS signal of compound **9** at Rt: 2.71 min [impurity, $(M+H)^+$=342]; HPLC-MS signal of *trans* compound **11** at Rt: 1.71 min [$(M+H)^+$=399]; HPLC-MS signal of *cis* compound **11** at Rt: 1.60 min [$(M+H)^+$=399].

a)

b)

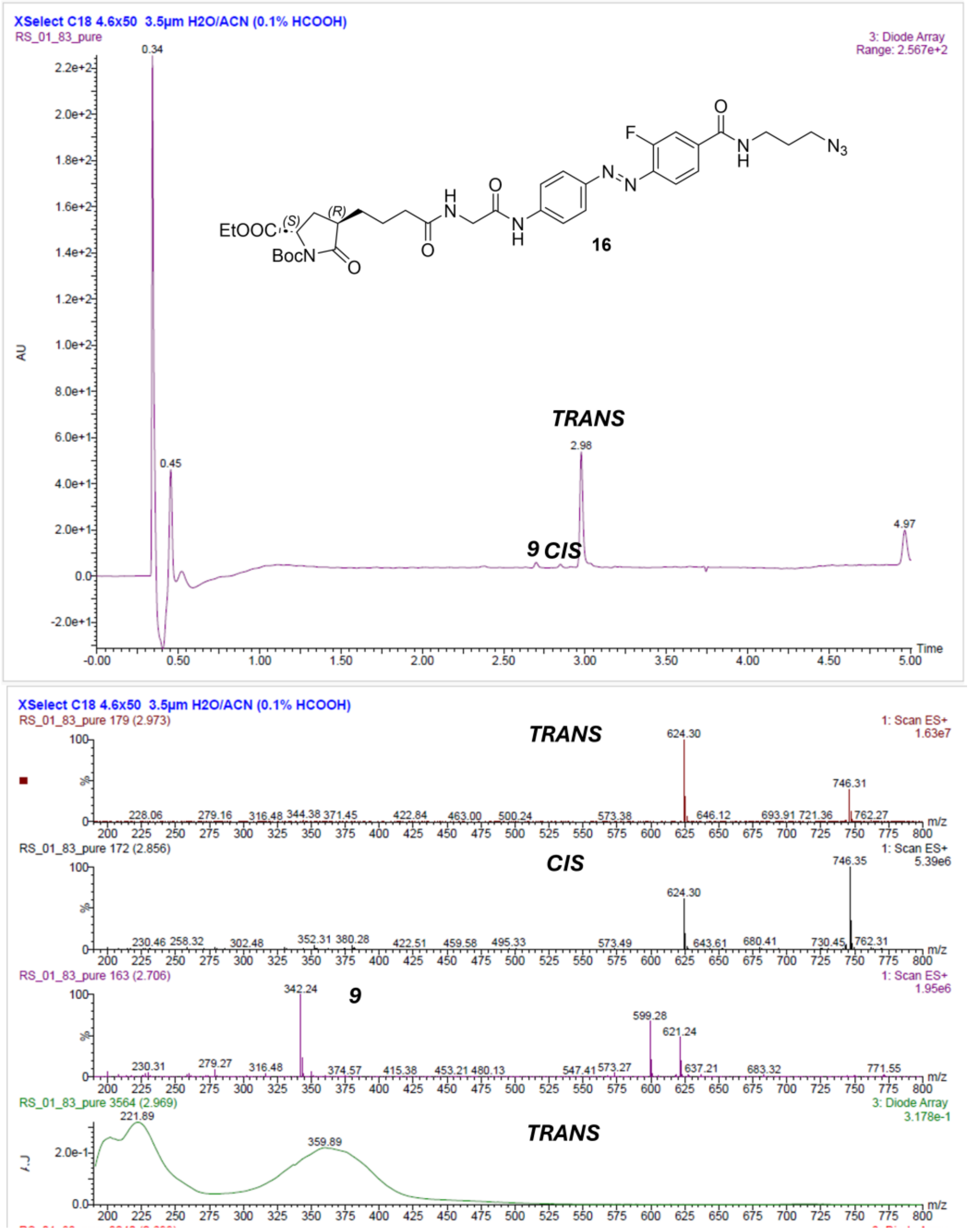

Figure S20. HPLC-PDA-MS of compound **16**. **a**) HPLC-PDA chromatogram of compound **16**. **b**) From bottom: HPLC-PDA trace of *trans* **16** (Rt: 2.98 min; λ: 360 nm);HPLC-MS signal of compound **9** at Rt: 2.71 min [impurity, $(M+H)^{+}$=342]; HPLC-MS signal of *cis* compound **16** at Rt: 2.86 min $[M+Na]^{+}$= 746, $[(M\text{-}Boc)+H]^{+}$= 624; HPLC-MS signal of *trans* **16** at Rt: 2.98 min $[M+Na]^{+}$= 746, $[(M\text{-}Boc)+H]^{+}$= 624.

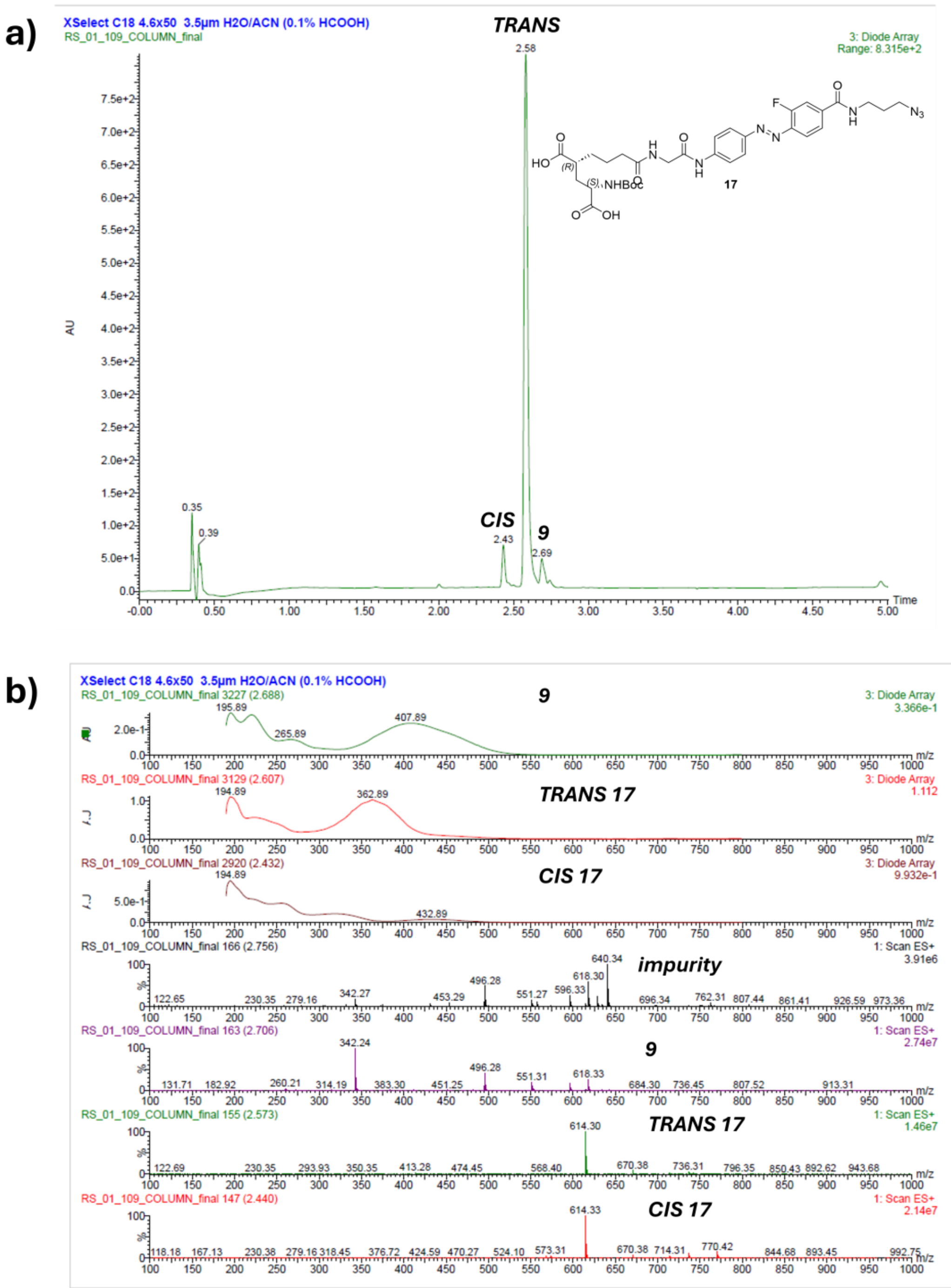

Figure S21. HPLC-PDA-MS of compound **17**. **a**) HPLC-PDA chromatogram of compound **17**. **b**) From top: HPLC-PDA trace of **9** (impurity, Rt: 2.69 min; λ: 408 nm); HPLC-PDA trace of *trans* **17** (Rt: 2.58 min; λ: 363 nm); HPLC-PDA trace of *cis* **17** (Rt: 2.43 min; λ: 433 nm); HPLC-MS signal of impurity at Rt: 2.77 min ; HPLC-MS signal of **9** [impurity, $(M+H)^+=342$]; HPLC-MS signal of *trans* compound **17** at Rt: 2.58 min $[M+Na]^+= 736$, $[(M\text{-}Boc)+H]^+= 614$; HPLC-MS signal of *cis* compound **17** at Rt: 2.43 min $[(M\text{-}Boc)+H]^+= 614$.

a)

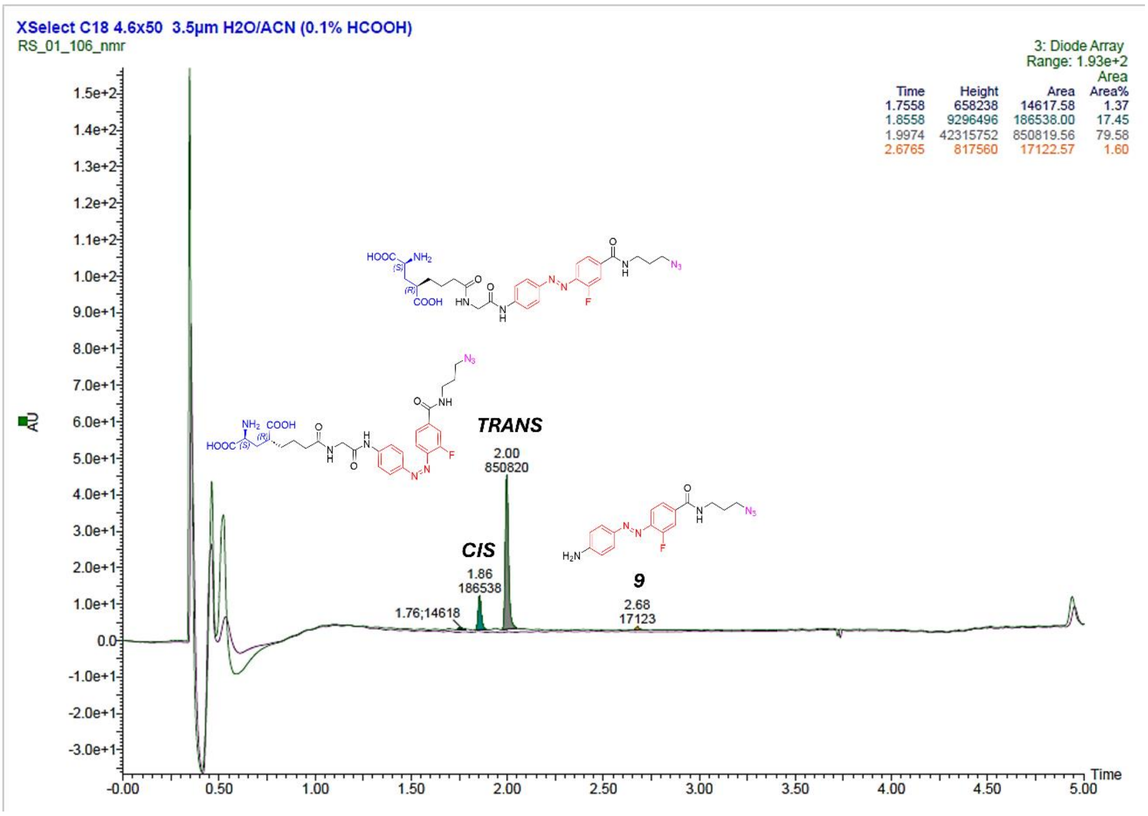
XSelect C18 4.6x50 3.5µm H2O/ACN (0.1% HCOOH)
RS_01_106_nmr
3: Diode Array
Range: 1.93e+2
Time Height Area Area%
1.7558 658238 14617.58 1.37
1.8558 9296496 186538.00 17.45
1.9974 42315752 850819.56 79.58
2.6765 817560 17122.57 1.60
TRANS
2.00
850820
CIS
1.86
186538
1.76;14618
9
2.68
17123
AU
Time

b)

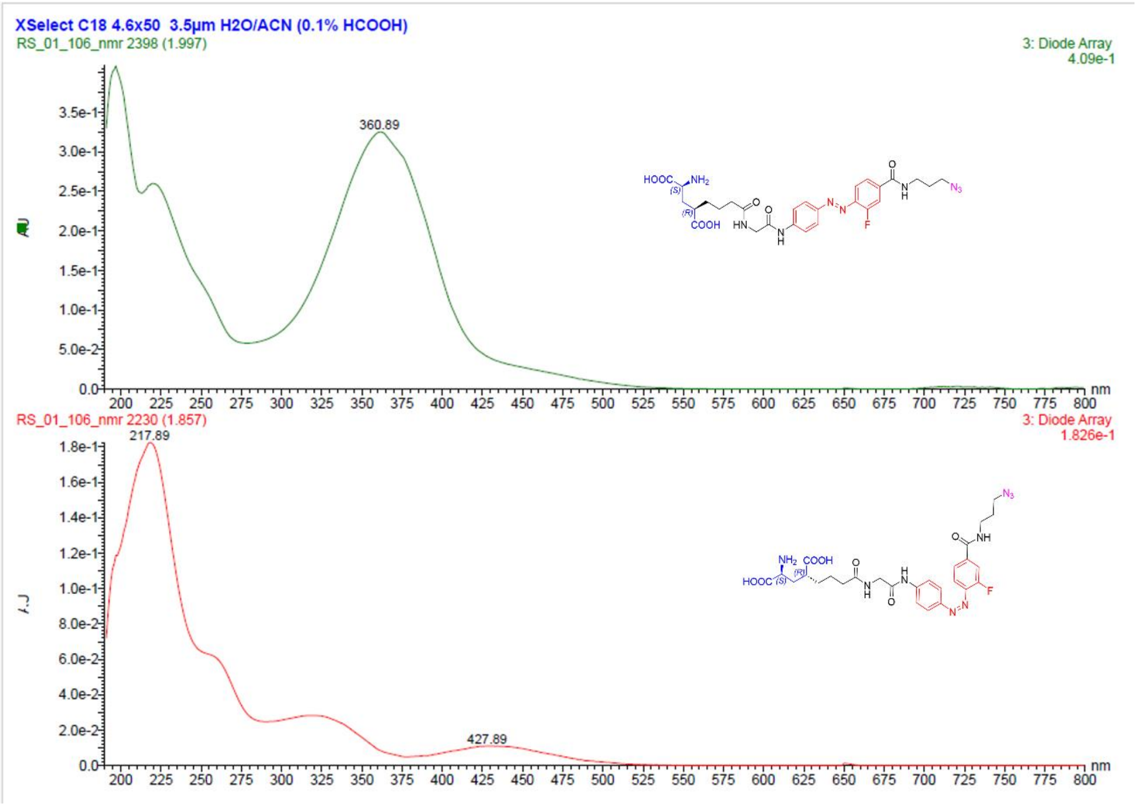
XSelect C18 4.6x50 3.5µm H2O/ACN (0.1% HCOOH)
RS_01_106_nmr 2398 (1.997)
3: Diode Array
4.09e-1
360.89
nm
RS_01_106_nmr 2230 (1.857)
3: Diode Array
1.826e-1
217.89
427.89
nm

c)

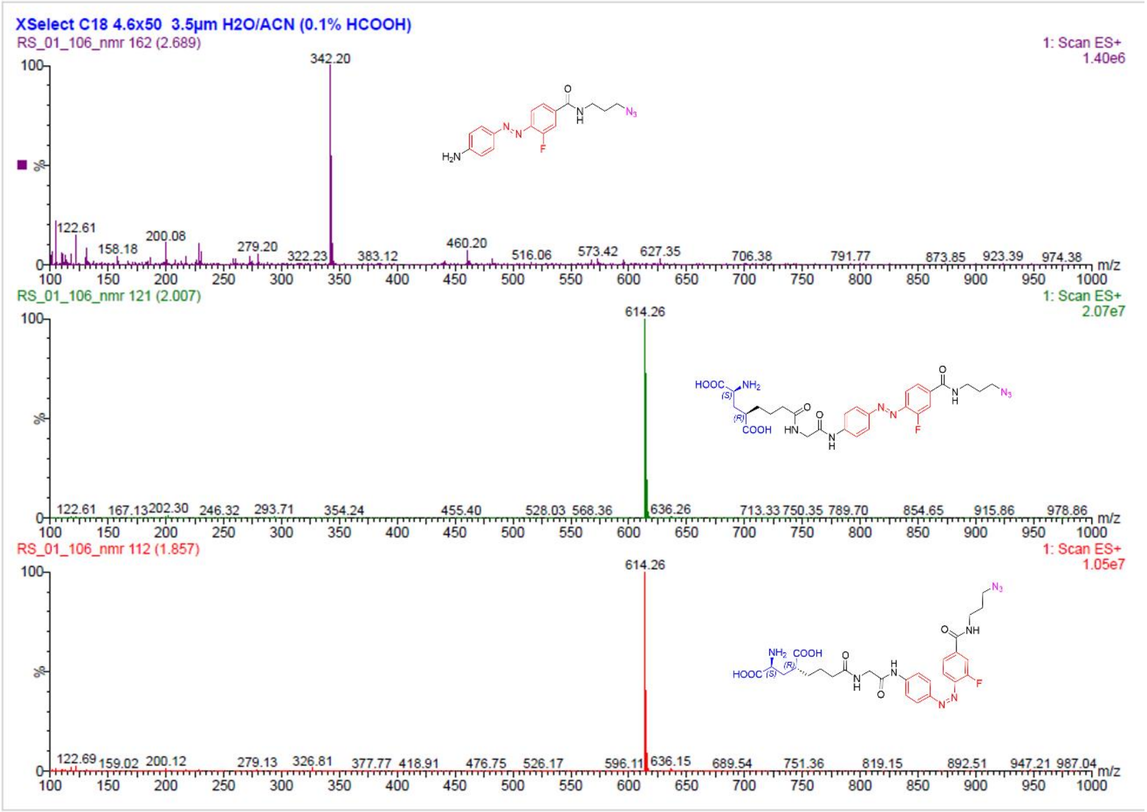

Figure S22. HPLC-PDA-MS of compound **18.** **a**) HPLC-PDA chromatogram of compound **18**. **b**) HPLC-PDA traces of *trans* **18** (Tr: 2.00 min, λ: 361 nm) and *cis* **18** (Tr: 2.00 min, λ: 428 nm). **c**) From top: HPLC-MS signals of **9** [impurity, $(M+H)^+$=342]; HPLC-MS signal of *trans* compound **18** at Rt: 2.00 min $[M+H]^+$= 614; HPLC-MS signal of *cis* compound **18** at Rt: 1.86 min [$(M+H)^+$= 614].

RS01106

Mass Spectrum (m/z 200-200)

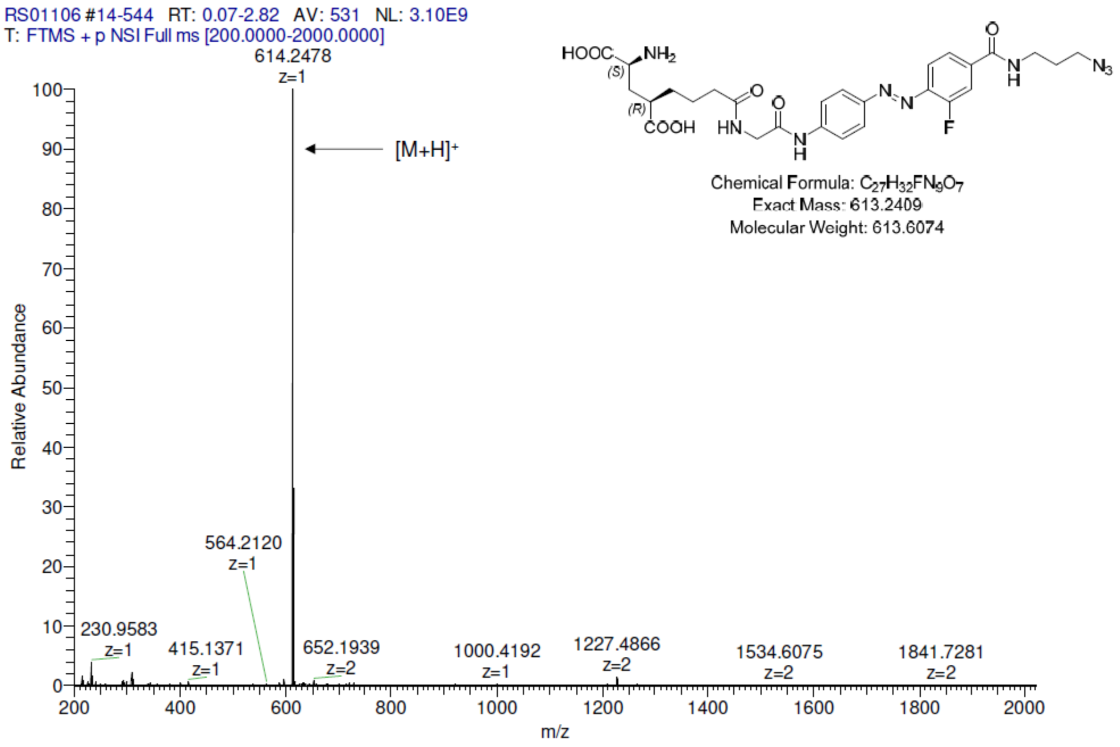

RS01106

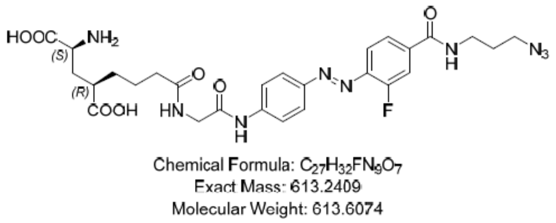

Elemental composition search on mass 614.25

m/z= 609.25-619.25

m/z	Theo. Mass	Delta (ppm)	RDB equiv.	Composition
614.24781	614.24815	-0.55	15.5	C 27 H 33 O 7 N 9 F

← [M+H]+

Figure S23. High resolution mass spectrum of compound **18.**

Photochemical characterization

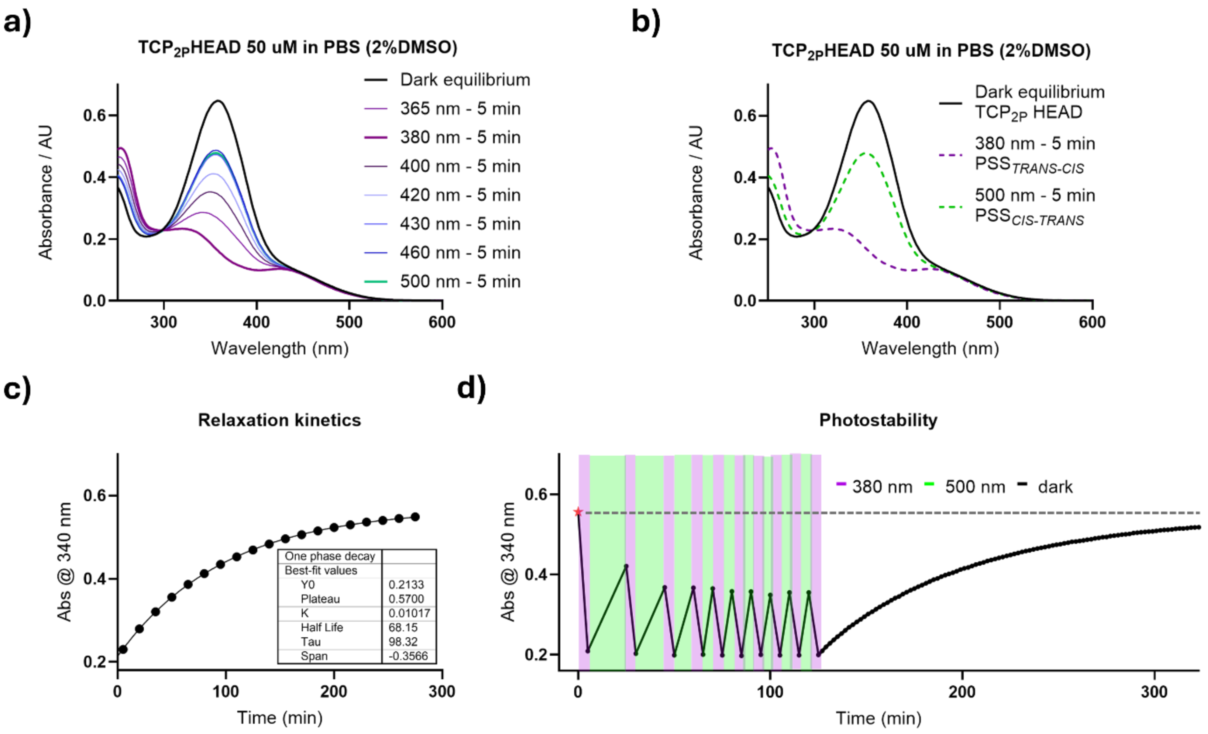

Figure S24. Photochemical characterization of compound 18. a. Absorption spectrum of compound **18**. Spectra were recorded with a UV-spectrophotometer using a 50 µM solution in PBS. **b**. The best rate of photoconversion from *trans* to *cis* is achieved by irradiating with 380 nm light for five minutes. The more thermodynamically stable *trans* isomer is recovered by irradiating for 5 minutes with 500 nm light or by thermal relaxation. **c**. The thermal back relaxation kinetics were studied by irradiating the cuvette with 380 nm light for five minutes and recording the absorbance at 340 nm in the dark every 15 minutes. One phase decay fit of the time course of the spontaneous switching from *cis* to *trans* for a 50 µM solution in PBS (2% DMSO) at absorption peak of 340 nm. $T_{1/2}$ = 68 min, tau = 98 min. **d.** Stability of compound **18** to ten cycles of irradiation. After the last cycle, compound **18** was left in the dark and it recovered its initial *trans* conformation. This indicates that several cycles of irradiation do not cause photodegradation.

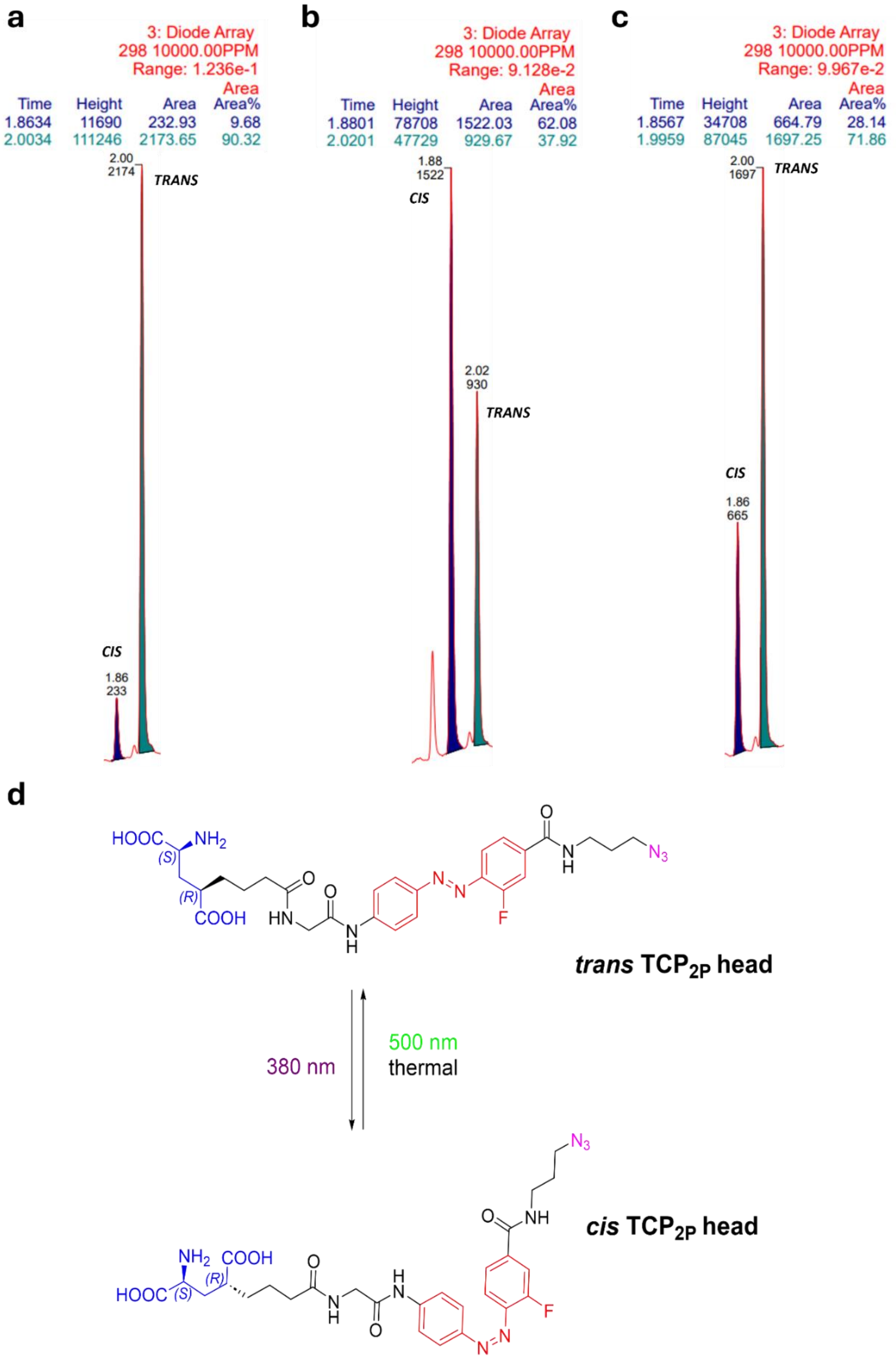

Figure S25. HPLC-PDA chromatogram of **TCP$_{2P}$ head** at 298 nm (isosbestic point). **a**. Benchtop condition. 90 % of *trans* and 10% of *cis* isomer. **b**. Photostationary state (PSS) after irradiating for five minutes with a 380 nm LED. 38% *trans* and 62% *cis*. **c**. Equilibrium between *trans* and *cis* isomers after irradiating for five minutes with a 500 nm LED. A recovery of 72 % of the *trans* isomer was observed. **d**. Schematic representation of TCP$_{2P}$ head *trans* and *cis* isomers.

Synthetic protocol for the preparation of TCP$_{2P}$

Head (**18**) and **tail** (**19**) (CAS: 1174157-65-3, commercially available) were combined to form **TCP$_{2P}$** compound using a “click” version of the Huisgen azide-alkyne 1,3-dipolar cycloaddition[7,8].).

Compound **18** (1 eq, 0.7 mg, 1.14 µmol), Cu_2O (2.40 eq, 0.4 mg, 2.74 µmol) and sodium ascorbate (4 eq, 0.9 mg, 4.56µmol) were weighted in a 0.5 mL Eppendorf, and the mixture was added of 30 µL of Milliq water and vortexed for two minutes. The tail (**19**) was weighted in an Eppendorf (1.3 mg) and dissolved in 50 µL of THF to obtain a 115 mM final solution. 11 µL of the tail 115 mM solution (0.3 mg, 1.25 µmol) were added to the head mixture that was then vortexed for 30 minutes.

The mixture was added of 74 uL of DMSO and vortexed for 10 seconds, then it was spinned to remove the precipitate. The clear orange solution (10 mM) was divided in aliquots of 10 µL that were stored at -80 °C.

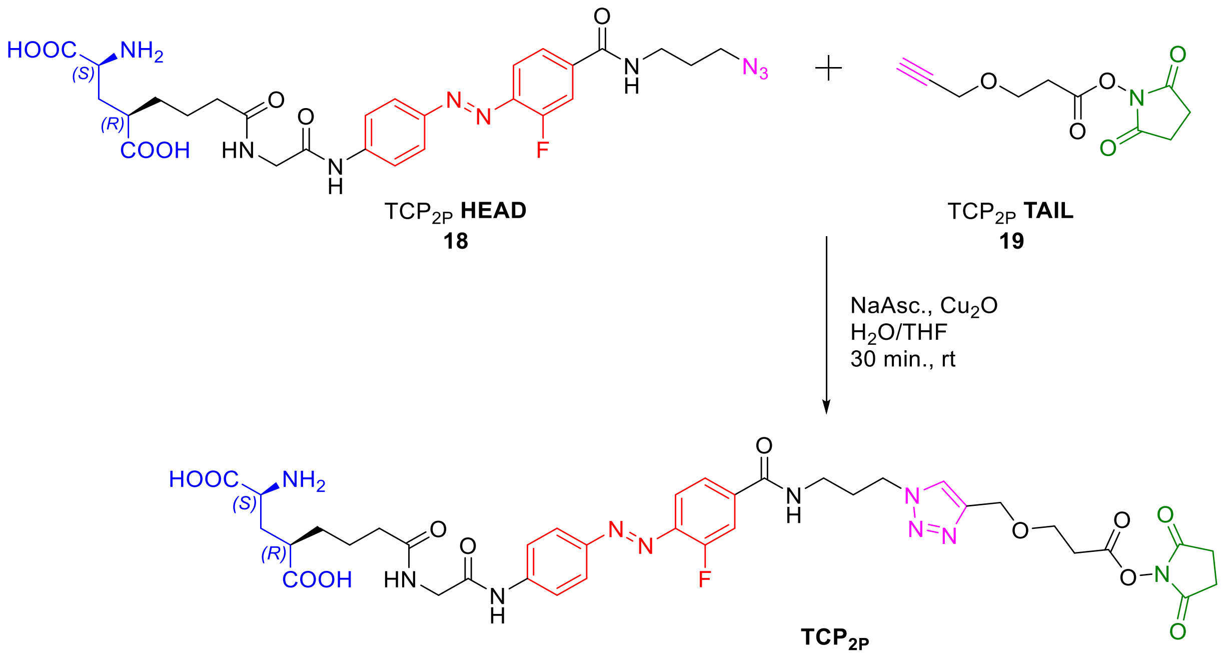

Scheme S2. Synthesis of TCP$_{2P}$ via Cu(I)-catalyzed azide alkyne 1,3-dipolar cycloaddition reaction.

Outcome of click reactions

Each batch of click allowed to obtain around 11 vials, each containing 10 uL solution in a theoretical 10 mM concentration of the final TCP$_{2P}$ compound. The outcome of each click was monitored by HPLC-MS. To the 10 µL solution, 50 µL of MeOH was added, and the final mixture was injected to verify the presence of the final compound. Figure S20a shows the HPLC-PDA chromatogram of the best click, while Figure S20b shows the HPLC-MS chromatogram of the final clicked compound.

a

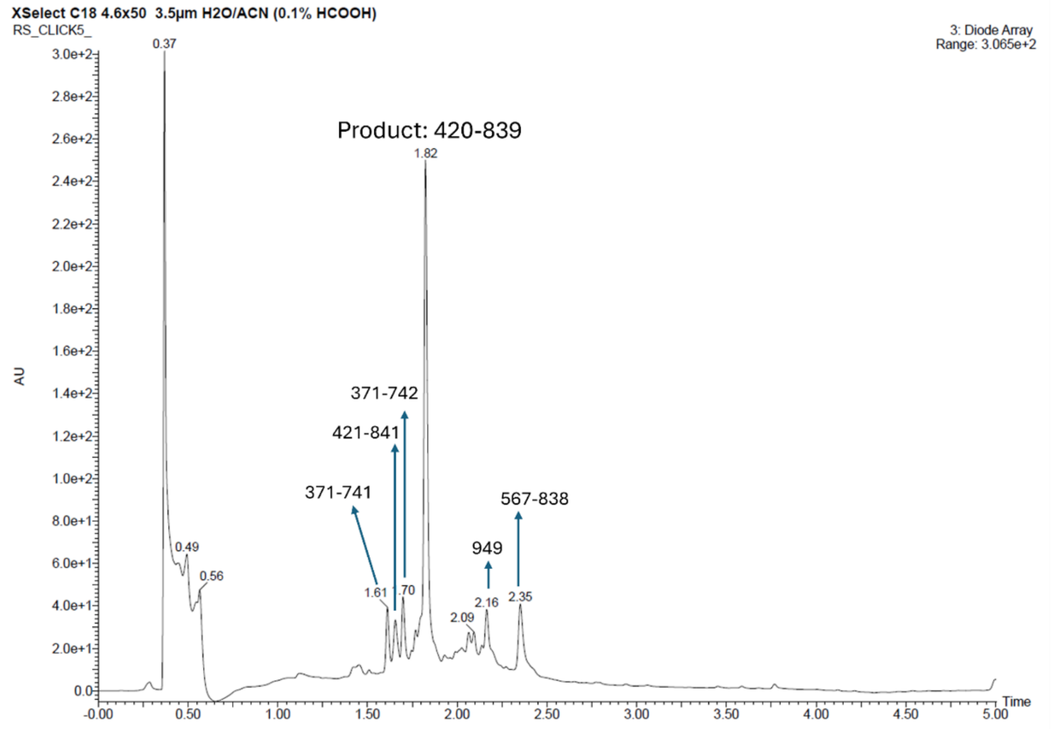

b

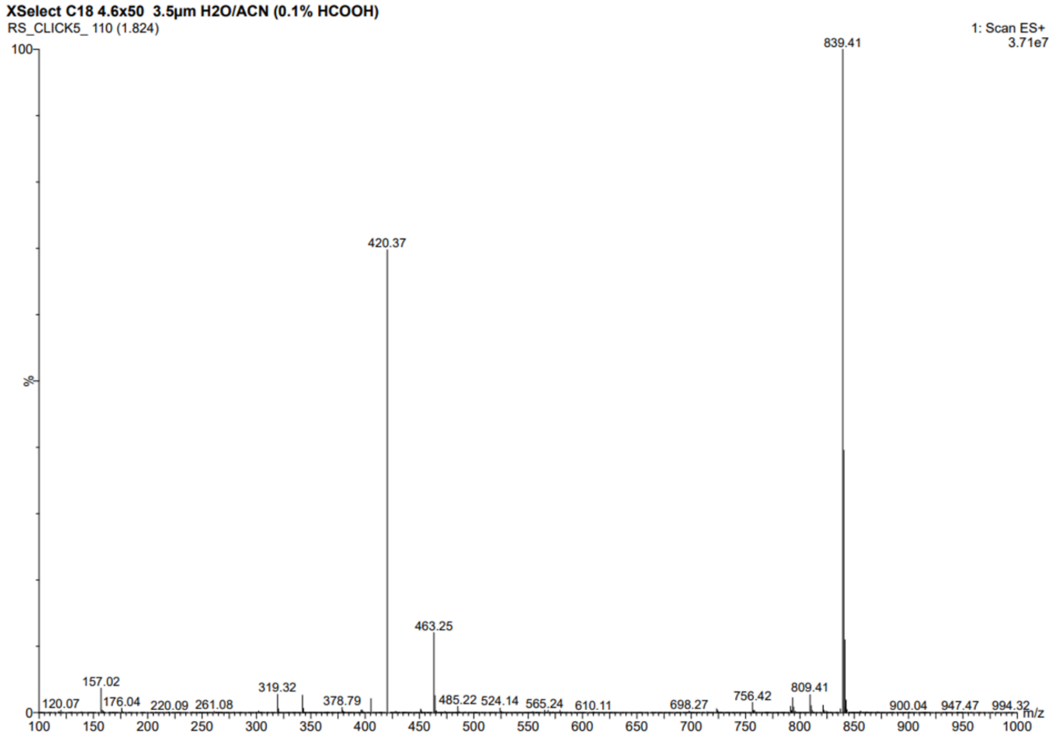

Figure S26. a. HPLC-PDA chromatogram of Click reaction, affording **TCP$_{2P}$**; **b.** HPLC-MS chromatogram of TCP-2P. Tr=1.82; $[M+H]^+$= 839

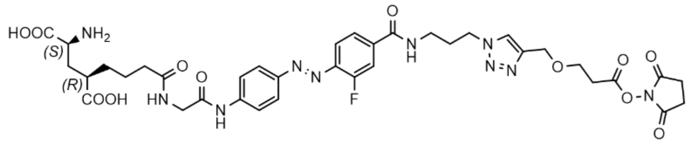

m/z: 838.30 (100.0%), 839.31 (41.0%), 840.31 (10.7%), 839.30 (3.7%), 841.31 (2.4%), 840.30 (1.5%)

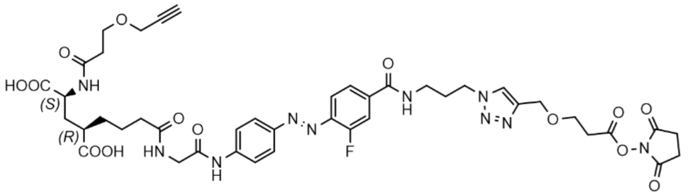

m/z: 948.34 (100.0%), 949.34 (50.2%), 950.35 (14.0%), 951.35 (3.5%), 950.34 (1.8%), 949.35 (1.1%)

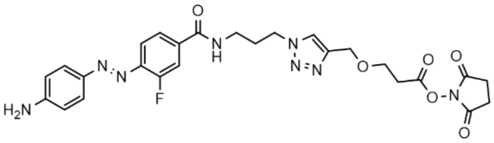

m/z: 566.20 (100.0%), 567.21 (28.7%), 568.21 (5.2%), 567.20 (3.0%)

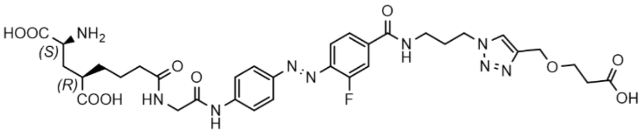

m/z: 741.29 (100.0%), 742.29 (39.9%), 743.29 (9.4%), 744.30 (1.5%)

m/z: 669.27 (100.0%), 670.27 (33.2%), 671.27 (7.9%), 670.26 (3.3%)

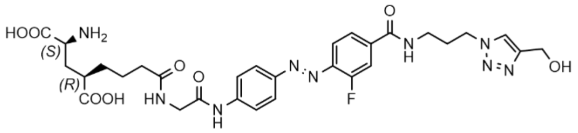

Figure S27: possible side-products from click 5.

As already reported by Izquierdo Serra *et Al.*[6], the main side-products result from the hydrolysis of the tail or the attachment of the hydrolysed tail on the amino group of the glutamate unit (Figure S21).

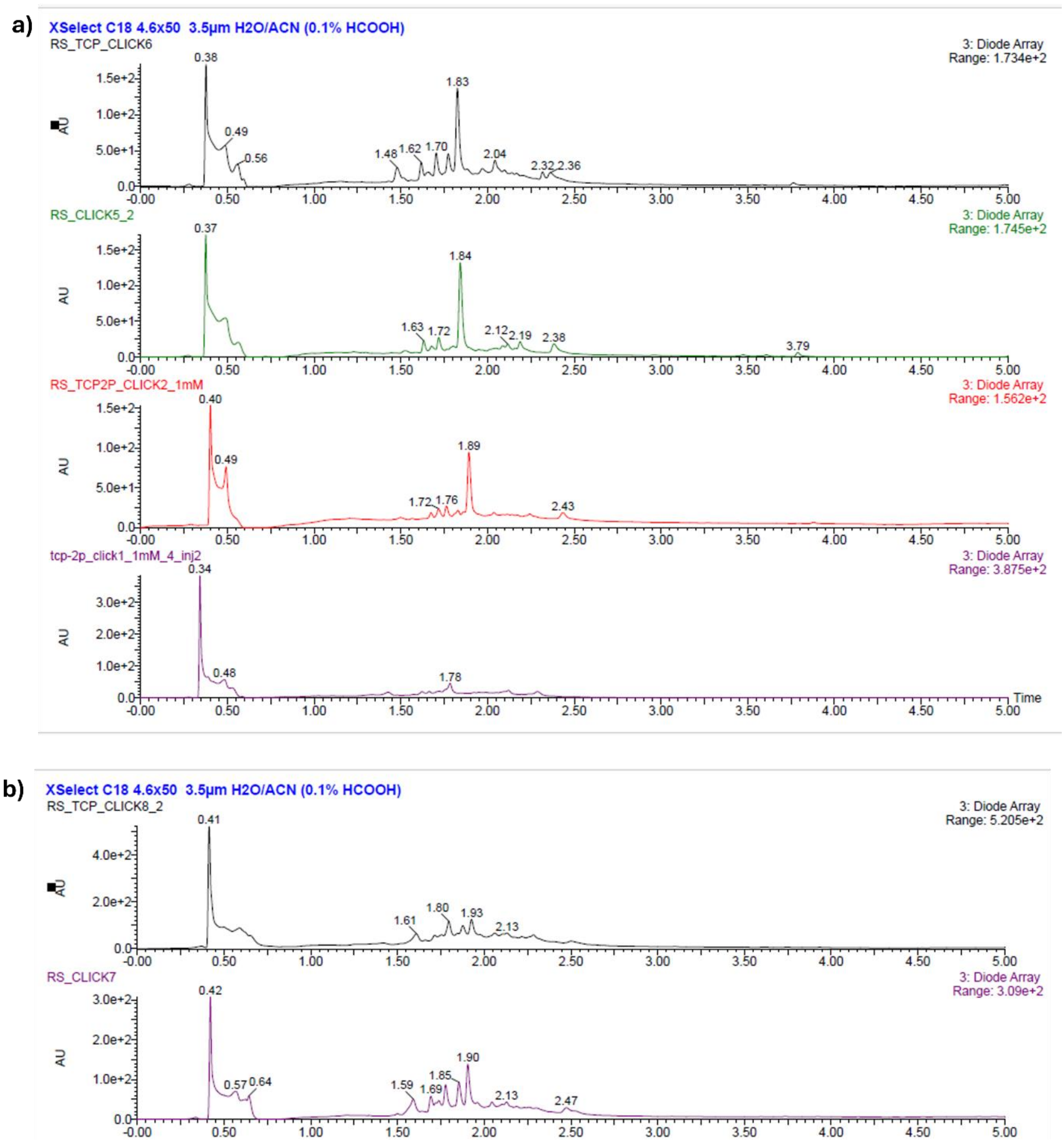

Figure S28. Outcome of click reactions. a) HPLC-PDA traces of successful click reactions. b) HPLC-PDA traces of unsuccessful click reactions, mainly because of the tendency of the tail reagent to hydrolyse with time.

Primary culture of hippocampal neurons

Procedures were performed in accordance with the European guidelines for animal care and use in research (EU directive 2010/63/EU and Spanish guidelines, Laws 32/2007, 6/2013, and RD 53/2013) and were approved by the Animal Experimentation Ethics Committee of the Barcelona Science Park (PCB, 21-000-PG). Sprague-Dawley rat pups (P 1-3) were euthanized by decapitation, both hippocampi of 3 pups were isolated and treated with 0.1 % trypsin in HBSS (10 min, 37ºC). Cells were plated on Poly-D-Lysine (PDL)-coated 16 mm coverslips (0.5-1 x 10^5 cells per coverspil) and incubated at 37°C, 5% CO2 for 1.5 hours (to allow cells to attach) in MEM supplemented with heat-inactivated FBS (5 %),

heat-inactivated HS (5 %), penstrep (10 UI/ml), L-glutamine (2 mM) and glucose (20 mM). Cells were cultured in Neurobasal A medium, supplemented with B-27 (5 %), penstrep (5 UI/ml), glutaMAX (0.5x) and glucose (15 mM). On the 3d day *in vitro* (DIV) culture was treated with 1 µM of Ara-C to prevent proliferation of microglial cells. Every 3-4 days 50% of the maintenance medium was exchanged for the fresh one. Electrophysiology experiments were performed on neurons older than 13 DIV.

Electrophysiological recordings

Whole-cell recordings were performed at room temperature using an EPC-10 amplifier (HEKA Elektronik, Germany) and Patch Master software (HEKA). For the experiments cells were places in a recording chamber and bathed in the external recording solution containing (mM): 150 NaCl, 3 KCl, 2 $MgCl_2$, 10 HEPES, 10 D-glucose and 2 $CaCl_2$; pH of the solution was adjusted to 7.35 - 7.40 with NaOH. Recording pipettes were pulled from borosilicate glass capillaries (Harvard Apparatus Ltd, USA) and had resistance 5-10 MOhms. Recording pipettes were filled with solution containing (mM): 115 K-gluconate, 10 KCl, 10 HEPES, 10 EGTA, 5 Mg-ATP, 0.5 Na-GTP; pH of the solution was adjusted to 7.3 with KOH.

Calcium imaging

Calcium imaging experiments were performed at room temperature, cells were bathed in external recording solution containing (mM): 150 NaCl, 3 KCl, 1 $MgCl_2$, 10 HEPES, 10 D-glucose and 2 $CaCl_2$; pH was adjusted to 7.35 - 7.4 with NaOH. Before the experiment cultured hippocampal neurons were incubated in PBS solution containing 10 µM of calcium indicator Oregon Green BAPTA-1, AM (OGB-1AM, Life Technologies) for 30 min at 37 °C and 5 % CO_2. After incubation neurons were washed several times in extracellular solution and transferred to the recording chamber for imaging.

Calcium imaging for simultaneous one-photon (1P) activation of TCP_{2P} was performed using an IX71 inverted microscope (Olympus) with a XLUMPLFLN 20XW, NA 1 water immersion objective (Olympus) and Patch Master software (HEKA). OGB-1AM was excited with 488 nm light for 100 ms with 1900 ms intervals using a Polychrome V monochromic light source (Till Photonics) equipped with a Xenon Short Arc lamp (Ushio) and a 505 nm dichroic beam splitter (Chroma Technology). Emission signal (526 nm) was filtered by a D535/40nm emission filter (Chroma Technology) and registered by a C9100-13 EM-CCD camera (Hamamatsu).

Calcium imaging for two-photon (2P) activation of TCP_{2P} was performed using Zeiss LSM 790 confocal microscope equipped with 2P Mai Tai DeepSee Ultrafast laser using 40XW, NA 1.2 water immersion objectives (Zeiss). OGB-1 AM was excited at 488 nm using Argon laser, light was collected using internal detector (500-530 nm range) and 488 mirror beam splitter.

TCP_{2P} administration and photostimulation in live cell imaging experiments

Before each experiment, neurons were incubated in 200 µM solution of TCP_{2P} for 4 min in the extracellular incubation solution containing (mM): 100 NaCl, 2.5 KCl, 10 D-glucose, 2.5 $CaCl_2$, 1 $MgCl_2$ 2.7 Na_2CO_3, 47.3 $NaHCO_3$ (pH 9). After incubation cells were washed with extracellular recording solution 3-4 times and bathed with extracellular recording solution during electrophysiological or calcium imaging experiments. In imaging experiments shortly before stimulation with 1P or 2P light cyclothiazide (50 µM) was added to the recording chamber to prevent desensitization of GluARs. 1P switching of TCP_{2P} during electrophysiological recordings was done with 380 nm and 500 nm light

(power densities 0.18 mW·cm^{-2} and 0.094 mW·cm^{-2}, respectively) applied by Polychrome V continuously during the time intervals indicated on the figures. 1P switching of TCP_{2P} during calcium imaging experiments was done with 380 nm and 500 nm light (power densities 0.01 mW·cm^{-2} and 0.07 mW·cm^{-2}, respectively) for 1500 ms with 500 ms intervals every 2 s. 2P activation of TCP_{2P} in most experiments was done with 760 nm light coming from Mai Tai laser. In the experiments studying the efficiency of TCP_{2P} activation by different wavelengths of 2P light 720 nm, 760 nm, 800 nm, 840 nm lights were tested (power densities 38 - 44 mW·cm^{-2}). MBS 690+ dichroic beam splitter was used to direct the pulsed laser light to the specimen.

Drugs

Stock solutions of CNQX, AP V and cyclothiazide were prepared using MilliQ water and later diluted in the extracellular solution till final concentration.

Data analysis

Data was analyzed using Igor Pro 6.05 (WaveMetrics), GraphPad Prism 9 (GraphPad) and ImageJ (Fiji). Results are represented as mean ± standard error of the mean (SEM). Statistical difference between groups was evaluated using Friedman, one-way Anova and Mann-Whitney tests; it was considered significant at the value of P below 0.05.

Supplementary results of *in vitro* testing

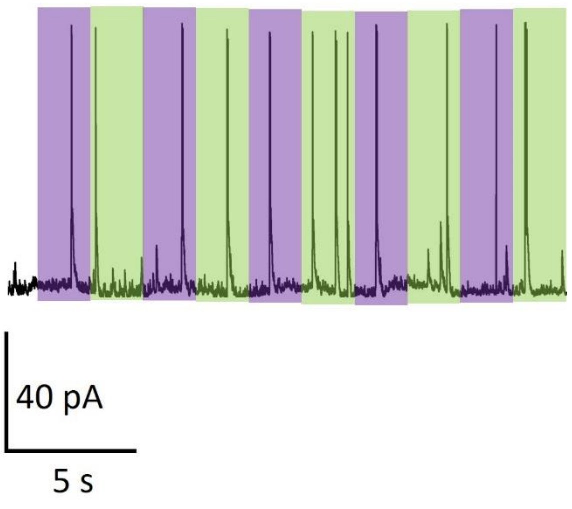

Figure S29. Representative electrophysiological recording from cultured hippocampal neurons that were not incubated in TCP_{2P}. Firing of the neuron is not affected by illumination with 380 nm or 500 nm light.

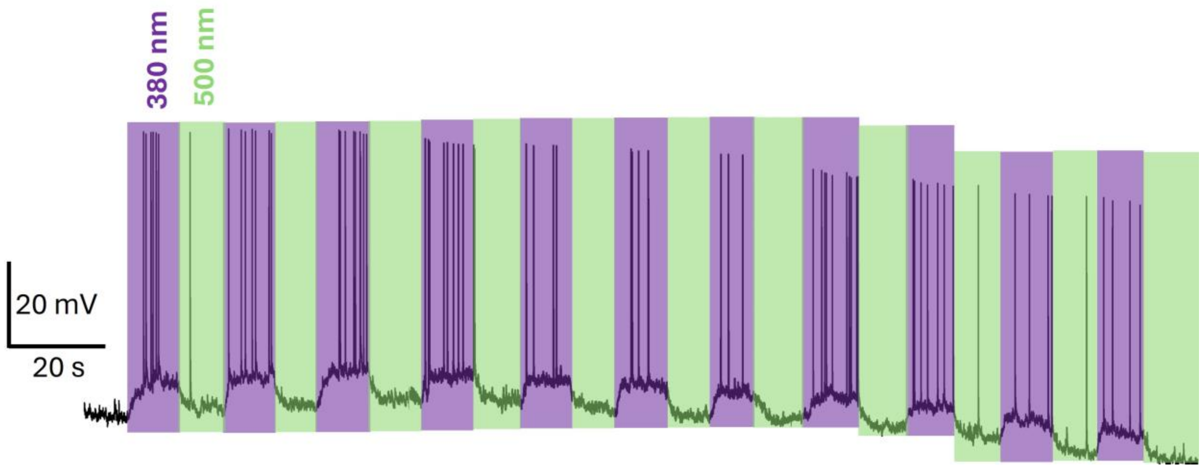

Figure S30. Representative fatigue on cultured hippocampal neurons incubated in TCP_{2P}. Repetitive cycles of irradiation do not display fatigue in agreement with Figure S24d.

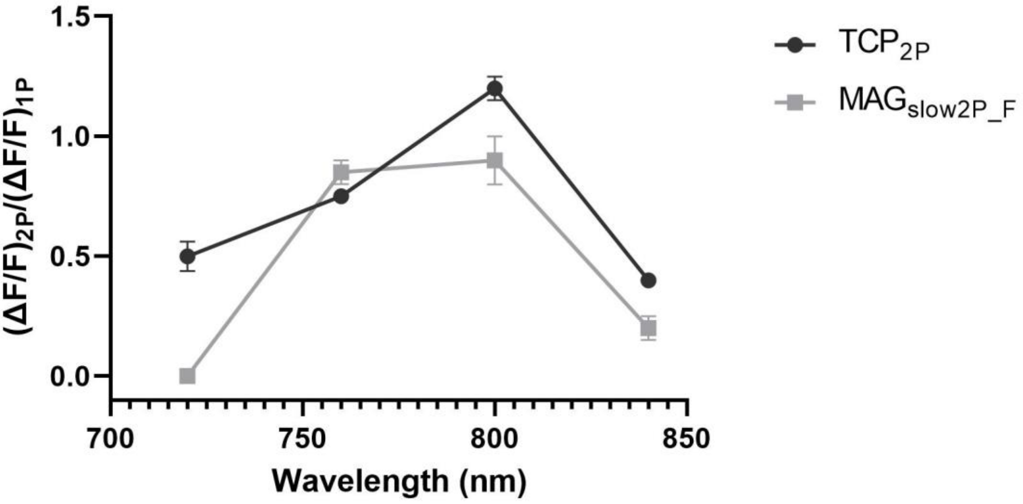

Figure S31. Ratios between 2PE and 1PE induced fluorescence calcium responses of TCP_{2P} (black line) and MAG_{slow2P_F} (gray line). Data for reference compound MAG_{slow2P_F} has been replotted from Cabré et al., 2019 (ref. 3).

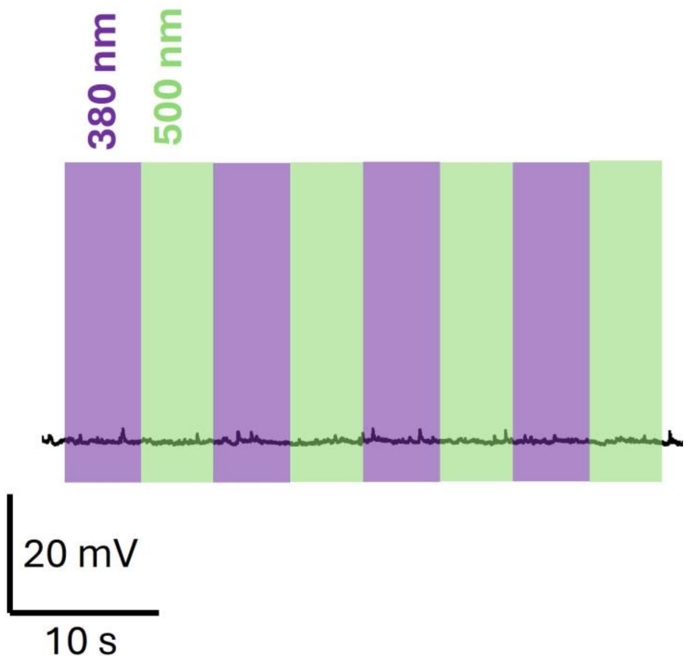

Figure S32. Representative recording from hippocampal neurons incubated for 4 min in 200 µM of the product of click reaction that contained all chemical components except the tail. Note the absence of any response to 380 nm or 500 nm illumination. No light-dependent firing was observed in 3 independent neurons.

Abbreviations

2PE: two-photon excitation

3PE: three photon excitation

AB: azobenzene

ACN: acetonitrile

AP: aqueous phase

APV: (2R)-amino-5-phosphonopentanoate

AU: arbitrary units

BOC: tert-butyloxycarbonyl

Cd3od: deuterated methanol

Cdcl3: deuterated chloroform

CNQX: 6-cyano-7-nitroquinoxaline-2,3-dione

Cu_2O: copper oxide (I)

D_2O: deuterated water

DCM: dichloromethane

d-dmso: deuterated dimethylsulfoxide

DIPEA: N,N-Diisopropiletilamina

DMAP: 4-Dimethylaminopyridine

DMF: dimethylformamide

DMSO: dimethylsulphoxide

DNQX: 6,7-dinitroquinoxaline-2,3-dione

EDC: 1-Ethyl-3-(3-dimethylaminopropyl)carbodiimide

EDG: electron donating group

EtOAc: ethyl acetate

EWD: electron withdrawing group

Fmoc: fluorenylmethoxycarbonyl protecting group

Glu: glutamate

GluAR: AMPA - α-amino-3-hydroxy-5-methyl-4-isoxazolepropionic acid receptor

GluKR: kainate receptor

GluNR: NMDA - *N*-methyl-D-aspartate receptor

GPCR: G-protein coupled receptor

H_2: molecular hydrogen

H_2O: water

HCl: hydrochloric acid

HCOOH: formic acid

HOBt: hydroxybenzotriazole

HPLC: high performance liquid chromatography

HRMS: high resolution mass spectrometry

Hz: hertz

IR: infrared

KOH: potassium hydroxide

LED: light emitting diode

LiHMDS: lithium bis(trimethylsilyl)amide

LiOH: lithium hydroxide

MAG: maleimide azobenzene glutamate

MeOH: methanol

$MgSO_4$: magnesium sulphate

MPE: multiphoton excitation

MS: mass

NaHCO3: sodium bicarbonate

$NaHSO_3$: sodium bisulphite

NaN_3: sodium azide

$NaNO_2$: sodium nitrite

NaOAc: sodium acetate

NaOAsc: sodium ascorbate

NaOH: sodium hydroxide

NMR: nuclear magnetic resonance

NS: not significant

Nu: nucleophile

OGB-1AM: BAPTA- Oregon Green™ 488 acetoxymethyl ester

OP: organic phase

PAI: Phthalimide-Azo-Iperoxo

PBS: phosphate-buffered saline

Pd: Palladium

PDA: PhotoDiode Array

PSS: photo stationary state

QDa: Quadrupole Dalton

Rt: retention time

rt: room temperature

SEM: standard error of the mean

TCP_{2P}: targeted covalent photoswitch two-photon

TCP 9: targeted covalent photoswitch 9

THF: tetrahydrofurane

TLC: thin layer chromatography

UV: ultraviolet

V_{hold}: holding potential

Bibliography

(1) Samanta, S.; Woolley, G. A. Bis-Azobenzene Crosslinkers for Photocontrol of Peptide Structure. *ChemBioChem* **2011**, *12* (11), 1712–1723. https://doi.org/10.1002/cbic.201100204.

(2) Yu, M.; Yu, Q.; Rutledge, P. J.; Todd, M. H. A Fluorescent "Allosteric Scorpionand" Complex Visualizes a Biological Recognition Event. *ChemBioChem* **2013**, *14* (2), 224–229. https://doi.org/10.1002/cbic.201200637.

(3) Cabré, G.; Garrido-Charles, A.; Moreno, M.; Bosch, M.; Porta-de-la-Riva, M.; Krieg, M.; Gascón-Moya, M.; Camarero, N.; Gelabert, R.; Lluch, J. M.; Busqué, F.; Hernando, J.; Gorostiza, P.; Alibés, R. Rationally Designed Azobenzene Photoswitches for Efficient Two-Photon Neuronal Excitation. *Nat Commun* **2019**, *10* (1), 907. https://doi.org/10.1038/s41467-019-08796-9.

(4) Volgraf, M.; Gorostiza, P.; Numano, R.; Kramer, R. H.; Isacoff, E. Y.; Trauner, D. Allosteric Control of an Ionotropic Glutamate Receptor with an Optical Switch. *Nat Chem Biol* **2006**, *2* (1), 47–52. https://doi.org/10.1038/nchembio756.

(5) Levitz, J.; Pantoja, C.; Gaub, B.; Janovjak, H.; Reiner, A.; Hoagland, A.; Schoppik, D.; Kane, B.; Stawski, P.; Schier, A. F.; Trauner, D.; Isacoff, E. Y. Optical Control of Metabotropic Glutamate Receptors. *Nat Neurosci* **2013**, *16* (4), 507–516. https://doi.org/10.1038/nn.3346.

(6) Izquierdo-Serra, M.; Bautista-Barrufet, A.; Trapero, A.; Garrido-Charles, A.; Díaz-Tahoces, A.; Camarero, N.; Pittolo, S.; Valbuena, S.; Pérez-Jiménez, A.; Gay, M.; García-Moll, A.; Rodríguez-Escrich, C.; Lerma, J.; de la Villa, P.; Fernández, E.; Pericàs, M. À.; Llebaria, A.; Gorostiza, P. Optical Control of Endogenous Receptors and Cellular Excitability Using Targeted Covalent Photoswitches. *Nat Commun* **2016**, *7* (1), 12221. https://doi.org/10.1038/ncomms12221.

(7) Hermanson, G. T. *Bioconjugate Techniques*; Elsevier, 2008. https://doi.org/10.1016/B978-0-12-370501-3.X0001-X.

(8) Agard, N. J.; Prescher, J. A.; Bertozzi, C. R. A Strain-Promoted [3 + 2] Azide−Alkyne Cycloaddition for Covalent Modification of Biomolecules in Living Systems. *J Am Chem Soc* **2004**, *126* (46), 15046–15047. https://doi.org/10.1021/ja044996f.
